# Structural basis for recognition of N-formyl peptides as pathogen-associated molecular patterns

**DOI:** 10.1038/s41467-022-32822-y

**Published:** 2022-09-05

**Authors:** Geng Chen, Xiankun Wang, Qiwen Liao, Yunjun Ge, Haizhan Jiao, Qiang Chen, Yezhou Liu, Wenping Lyu, Lizhe Zhu, Gydo C. P. van Zundert, Michael J. Robertson, Georgios Skiniotis, Yang Du, Hongli Hu, Richard D. Ye

**Affiliations:** 1 Kobilka Institute of Innovative Drug Discovery, School of Medicine, The Chinese University of Hong Kong, Shenzhen, Guangdong 518172 China; 2 School of Life Sciences, University of Science and Technology of China, Anhui, 230026 China; 3 Shenzhen Bay Laboratory, Shenzhen, Guangdong 518055 China; 4 Warshel Institute for Computational Biology, School of Medicine, The Chinese University of Hong Kong, Shenzhen, Guangdong 518172 China; 5 Schrödinger, New York, NY 10036 USA; 6 Department of Molecular and Cellular Physiology, Stanford University School of Medicine, Stanford, CA 94305 USA; 7 Department of Structural Biology, Stanford University School of Medicine, Stanford, CA 94305 USA

**Keywords:** Cryoelectron microscopy, Antimicrobial responses, G protein-coupled receptors

## Abstract

The formyl peptide receptor 1 (FPR1) is primarily responsible for detection of short peptides bearing N-formylated methionine (fMet) that are characteristic of protein synthesis in bacteria and mitochondria. As a result, FPR1 is critical to phagocyte migration and activation in bacterial infection, tissue injury and inflammation. How FPR1 distinguishes between formyl peptides and non-formyl peptides remains elusive. Here we report cryo-EM structures of human FPR1-Gi protein complex bound to *S. aureus*-derived peptide fMet-Ile-Phe-Leu (fMIFL) and *E. coli*-derived peptide fMet-Leu-Phe (fMLF). Both structures of FPR1 adopt an active conformation and exhibit a binding pocket containing the R201^5.38^XXXR205^5.42^ (RGIIR) motif for formyl group interaction and receptor activation. This motif works together with D106^3.33^ for hydrogen bond formation with the N-formyl group and with fMet, a model supported by MD simulation and functional assays of mutant receptors with key residues for recognition substituted by alanine. The cryo-EM model of agonist-bound FPR1 provides a structural basis for recognition of bacteria-derived chemotactic peptides with potential applications in developing FPR1-targeting agents.

## Abstract (web summary)

Detection of invading bacteria is key to immunity. Here, the authors report cryo-electron microscopy structures of agonist-bound formyl peptide receptor 1 (FPR1), that reveal structural basis for recognition of bacteria-derived formyl peptides.

## Introduction

G protein-coupled receptors (GPCRs) serve important physiological functions upon their activation by binding ligands of various chemical natures. With >800 genes in the human genome^1^, GPCRs constitute the largest family of membrane proteins as well as the largest cohort of potential drug targets in humans. Although many well-characterized GPCRs bind endogenous ligands including neurotransmitters, hormones and chemokines^2^, other GPCRs serve as biosensors for exogenous ligands such as photons, odorants, tastants, natural products and their metabolites^3^. The formyl peptide receptors (FPRs) belong to the Family A GPCRs of about 350 amino acids that bind peptides with an N-formylated methionine (fMet)^4^, a prominent feature of protein synthesis in bacteria and mitochondria. N-formyl peptides serve as a pathogen-associated molecular pattern (PAMP)^5,6^ for innate immunity against invading bacteria^7–9^. Published studies have shown that FPR1 and FPR2 can distinguish between peptides carrying fMet and those without the N-terminal fMet, mounting an immune response to selected pathogens while sparing commensal microbiota^10^. The shortest full agonist for FPRs is the tripeptide fMet-Leu-Phe (fMLF) from *E. coli*^11^, which activates FPR1 by coupling to the heterotrimeric Gi proteins^12,13^, inducing phagocyte chemotaxis, granule release and superoxide generation through a series of concerted actions leading to elimination of the invading microorganisms^7,8^. FPRs are also known for recognition of mitochondria-released formyl peptides that serve as damage-associated molecular patterns, thereby contributing to phagocyte infiltration to injured tissues and clearance of cell debris^14^.

To understand the structural basis of formyl peptide recognition, early studies focused on sequence comparison and functional characterization. In humans, the FPR gene family encodes 3 receptors, namely FPR1, FPR2 and FPR3. FPR1 is the primary receptor in phagocytes for detection of N-formyl peptides, whereas FPR2 (69% identical to FPR1 in amino acid sequence) binds a variety of ligands including not only formyl peptides but also annexin A1, serum amyloid A and lipoxin A_4_ that do not contain an fMet. Moreover, FPR2 mediates both inflammatory and anti-inflammatory functions, suggesting ligand-dependent biased signaling^15,16^. FPR3 has no strong preference for formyl peptides^17^. Previous studies employing biochemical and mutagenic approaches^18^ and more recent studies using molecular dynamics simulations^19,20^ reported that R205^5.42^ of FPR1 interacts directly with the N-formyl group. However, an arginine in this position has also been found in other Family A GPCRs, arguing against the specificity of such an interaction.

In this work, the FPR1 structures in complex with Gi proteins and two of the bacterial formyl peptides are determined using cryo-EM. The tripeptide fMLF is an *E. coli*-derived formyl peptide, and the tetrapeptide fMet-Ile-Phe-Leu (fMIFL) is an *S. aureus*-derived formyl peptide^21^. Both peptides exhibit high binding affinity and potency at FPR1 (≤5 × 10^−10^ M in binding and chemotaxis assays). The cryo-EM structures lead to the identification of a unique R201^5.38^XXXR205^5.42^ (RXXXR) motif that is critical for formyl peptide recognition and receptor activation. R201^5.38^ and R205^5.42^, together with D106^3.33^, play a critical role in the recognition of the formyl group and fMet side chain, in part through stabilization of the FPR1 binding pocket. Multiple hydrogen bonds and hydrophobic clusters work together to promote formyl peptide binding. In comparison, a non-formyl peptide of the same sequence (Met-Leu-Phe) has a different binding pose to FPR1 as shown by molecular docking. The structural information illustrates how FPR1 recognizes short formyl peptides using a well-defined ligand binding pocket that has common and distinct features compared with the recently identified FPR2 ligand binding pocket^22,23^.

## Results

### Cryo-EM structure of the FPR1-Gi complex

**Fig. 1:**
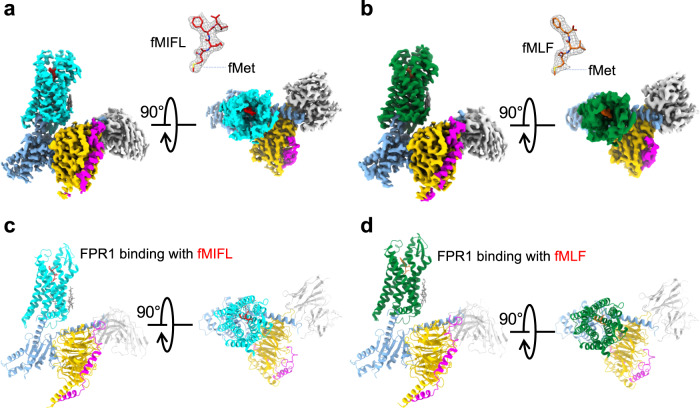
EM density map and overall structure of FPR1-Gi-scFv16 bound to N-formyl peptides. **a, b** Side view and extracellular view of the 3D cryo-EM density map of FPR1-Gi-scFv16 bound to fMIFL (**a**) and fMLF (**b**). The position of the N-terminal fMet is marked (insets). **c**, **d** Side view and extracellular view of the overall structure in cartoon representations. FPR1 bound to formyl peptide is colored in cyan (**c**, fMIFL) or green (**d**, fMLF), respectively. Gαi, Gβ1, Gγ2, scFv16 are colored in marine blue, yellow, magenta, and gray, respectively.

**Fig. 2:**
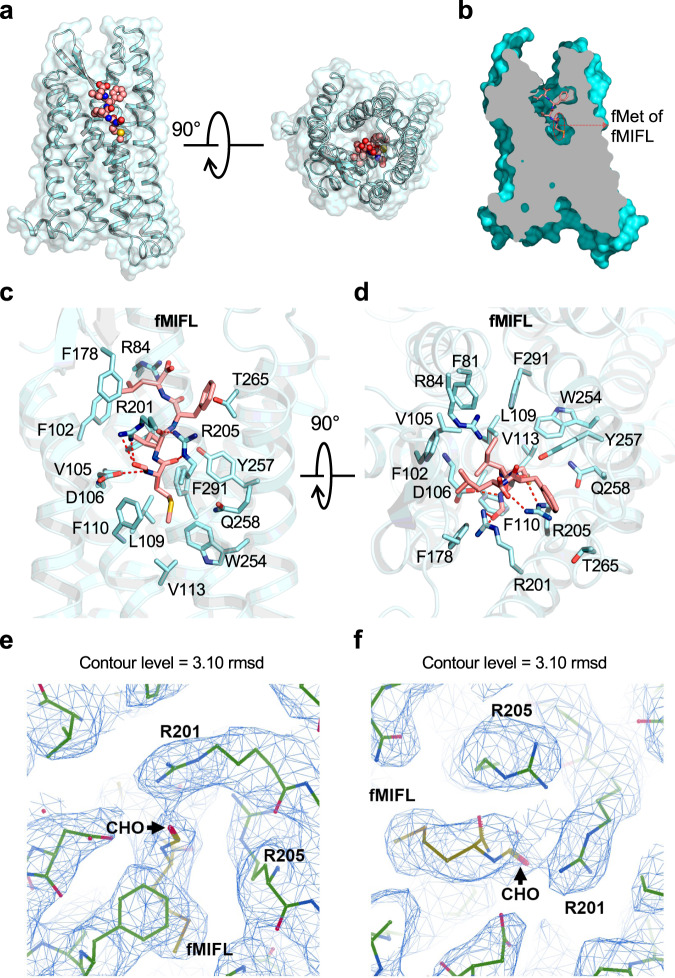
Ligand binding mode of FPR1 to fMIFL. **a** Side view (left) and extracellular view (right) of the FPR1-fMIFL structure. The receptor is shown as surface and cartoon, colored in cyan. The ligand fMIFL is shown as sphere with carbons in pink. **b** Slab view (light gray) of the binding cavity of fMIFL in FPR1. fMIFL assumes an N-terminus-in pose circled in red dashed line. **c** Side view of the binding pocket of FPR1-fMIFL structure. The receptor is shown as cartoon and colored in marine blue. The ligand fMIFL is shown as licorice with carbons in pink. Hydrogen bonds formed of R201^5.38^XXXR205^5.42^ motif with the N-formyl group, carbonyl groups of fMet in fMIFL, indicated in dash line. The residues of FPR1 within 4.5 Å to the atoms of fMIFL are shown in cyan licorice. **d** Extracellular (top) view of the FPR1-fMIFL structure. Red dashed lines indicate polar interactions between D106^3.33^, the R201^5.38^XXXR205^5.42^ motif, and fMet in fMIFL. **e**, **f** Local density map of the ligand fMIFL and residues of FPR1 nearby the formyl group (CHO), viewed from two different angles.

The FPR1-Gi-scFv16 complex bound to the *S.aureus*-derived tetrapeptide fMIFL was prepared (Supplementary Fig. 1, 2) and its structure was determined by cryo-EM to an overall resolution of 2.8 Å (Fig. 1; Supplementary Fig. 3, 4 and Supplementary Table 1). The antibody fragment scFv16^24^ was included to stabilize the structure of the receptor-G protein complex. The ligand binding pocket of FPR1 was surrounded by transmembrane (TM) helices 2, 3, 5, 6, with minor involvement of TM7 (Fig. 1). The tetrapeptide ligand fMIFL assumes a pose with its N-terminus inserted into the binding pocket (Fig. 2a, b). The N-formyl-Met (fMet) is surrounded by several charged residues (D106^3.33^, R201^5.38^ and R205^5.42^), placed in such a way that hydrogen bonds may form between the side chains of these amino acids and the nitrogen atom of fMet, the oxygen atom of the formyl group (CHO) and the oxygen atom of the carbonyl group on fMet, respectively (Fig. 2c, d). The density linkage between the formyl group and the side chain of R201^5.38^ can be seen at a contour level of 3.10 rmsd (Fig. 2e, f), favoring polar interaction between the formyl oxygen and R201^5.38^. In comparison, the side chain of R205^5.42^ is closer to the carbonyl oxygen of fMet for hydrogen bond formation. In addition to the polar interactions, fMet is surrounded by a hydrophobic pocket formed by L109^3.36^, F110^3.37^, V113^3.40^, W254^6.48^, and Q258^6.52^ (Fig. 2c, d). The Ile at position 2 (I2) is surrounded by a hydrophobic environment formed by F81^2.60^, V105^3.32^, and F291^7.43^. As for Phe (F3), its arene ring forms hydrophobic interaction with T265^6.59^. The C-terminal Leu (L4) is surrounded by a hydrophobic cap formed by R84^2,63^, F102^3.29^, F178^ECL2^.

Previously published studies using site-directed mutagenesis^18^ and molecular docking^19,20^ identified R205^5.42^ for interaction with the N-formyl group. Since most of these studies were conducted using the tripeptide fMLF, we further obtained the cryo-EM structure of the fMLF-bound FPR1-Gi-scFv16 complex (Fig. 1). In this structure, the fMLF uses the same binding pocket as fMIFL and its N-formyl oxygen forms hydrogen bond with R201^5.38^ while the carbonyl oxygen of M1 forms hydrogen bond with R205^5.42^ (Supplementary Fig. 5). Like fMIFL, the L2 of fMLF is in a hydrophobic environment formed by F81^2.60^, V105^3.32^, and F291^7.43^. The C-terminal Phe (F3) of fMLF forms a hydrophobic interaction with F102^3.29^, T265^6.59^, and I268^ECL3^. With both ligands, the bottom of the binding pocket is composed with a hydrophobic environment involving L109^3.36^, F110^3.37^, V113^3.40^, W254^6.48^, and Q258^6.52^.

**Fig. 3:**
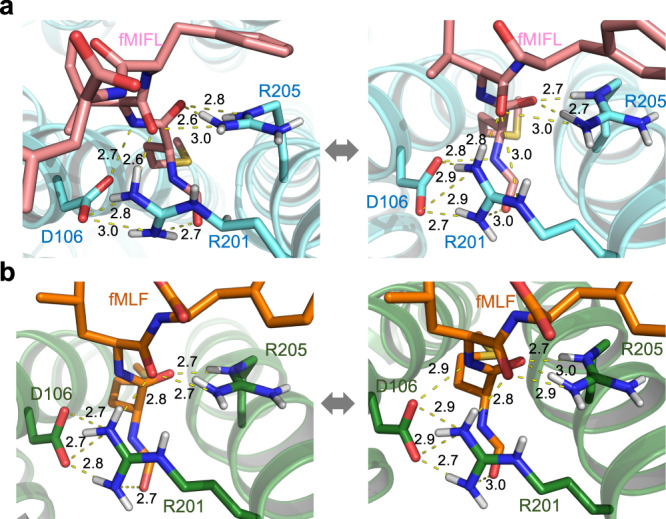
MD simulation of the Cryo-EM models. Two representative hydrogen-bonds networks characterized by either minimizing the average donor-acceptor (D–A) distance or maximizing the number of concurrent hydrogen-bonds from the conformation ensemble of the whole 3-µs trajectories of FPR1-Gi-scFv16 bound with fMIFL (**a**) and fMLF (**b**). Both representatives show that the formyl group is recognized by the D106^3.33^-R201^5.38^-R205^5.42^ motif, in particular the salt bridge between D106^3.33^ and R201^5.38^ stabilized the side-chain orientation of R201^5.38^. The residues D106^3.33^-R201^5.38^ directly recognizes the formyl-O of fMLF (or fMIFL), as both the short-range H-bond (left) and the long-range electrostatic attraction (right) could be offered by the positively charged guanidino group of R201^5.38^ during the thermodynamics fluctuation. The yellow dashed lines indicate distance shorter than 3 Å. Source data are provided as a Source data file.

Our modeling of the cryo-EM structures of FPR1 indicates that D106^3.33^ is in proximity to fMet for possible hydrogen bond formation (Fig. 2c, d). However, Asp is deprotonated under physiological conditions, hence preventing the formation of hydrogen bond with the oxygen atom in the formyl group. There remains the possibility of hydrogen bond formation between D106^3.33^ and the nitrogen atom of the amide group in fMet1 and Ile2. To test this possibility, we performed three independent 1-µs MD simulations to assess the stability of binding pose of fMIFL (Fig. 3a) and fMLF (Fig. 3b) to FPR1 involving D106^3.33^. The trajectory analysis shows that the overall conformation of the complex is very stable (Supplementary Fig. 6c, d). On the fMet1 of fMIFL, several hydrogen bonds may contribute to the recognition of fMIFL through interaction with the D106^3.33^-R201^5.38^-R205^5.42^ motif (Fig. 3a; Supplementary Fig. 6). Among these, the occupancy of the two hydrogen bonds (R205-NE⋯FME-O and R201-NH2⋯I2-O) are >95%. On fMet1, the formyl oxygen (FME-O1) and carbonyl oxygen (FME-O) also form hydrogen bonds with R201-NH1 and R205-NH2, respectively. In addition to the nitrogen atom of the amide group in fMet1, the nitrogen atom of Ile2 may form hydrogen bond with either D106-OD1 or D106-OD2. Besides, a stable salt bridge is observed between D106^3.33^ and R201^5.38^. Two representative hydrogen bond networks characterized by either minimizing the average D–A distance or maximizing the number of concurrent hydrogen bonds show that the salt bridge contributes to stabilization of the side-chain orientation of R201^5.38^. The same hydrogen bond networks were also observed in the binding of the tripeptide fMLF to FPR1 (Fig. 3b; Supplementary Fig. 5, Supplementary Fig. 6b, d). To further evaluate the ligand binding positions in FPR1, we applied GemSpot^25^, a tool for computational docking of ligands into cryo-EM densities, to obtain the binding poses of both fMIFL and fMLF. We observe good match between the high-scoring GemSpot poses (top-5 poses) and our proposed models, including the positions of the N-formyl oxygen in fMIFL and fMLF (Supplementary Fig. 7).

### Docking analysis for binding of formyl peptides, non-formyl peptides and non-peptide ligands

**Fig. 4:**
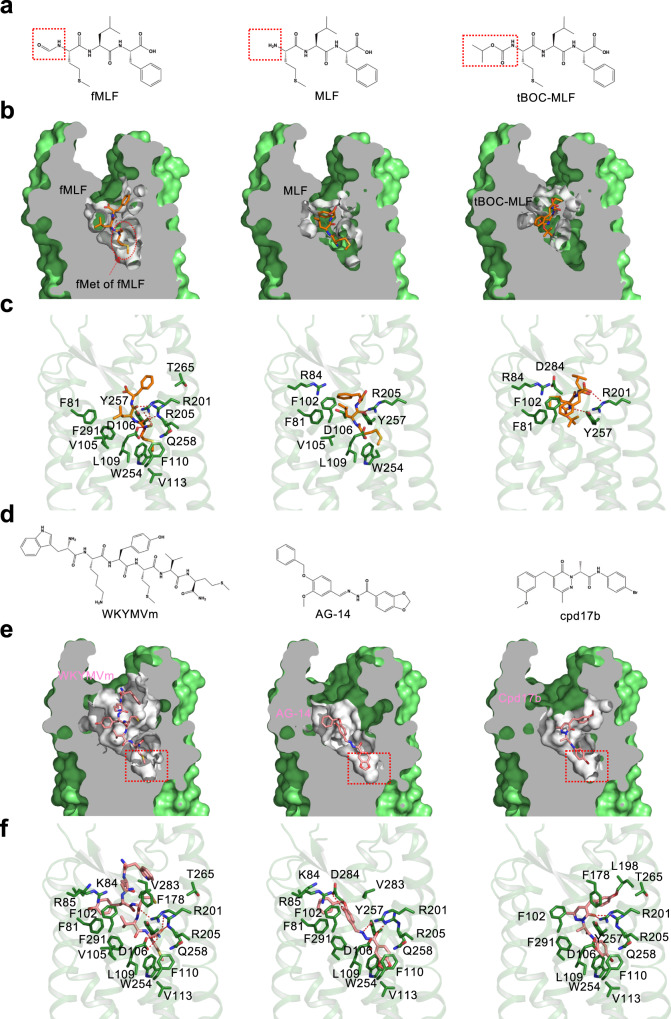
Binding poses of fMLF, non-formyl analogs and small molecule ligands to FPR1. **a** Chemical structure of fMLF and its non-formyl analogs. **b** Slab views of the binding pocket of fMLF (left, cryo-EM model), MLF (middle, docking model), and tBOC-MLF (right, docking model) in FPR1, respectively. The ligands are displayed in licorice with carbon in orange. The binding pocket is highlighted in white. **c** Molecular interaction of bound fMLF (left), MLF (middle), and tBOC-MLF (right) with the FPR1 binding pocket. **d** Chemical structures of WKYMVm, AG-14 and Compound 17b (Cpd 17b). **e** Slab views of the binding pocket of WKYMVm, AG-14 and Cpd 17b, all from docking models. **f** molecular interaction of bound WKYMVm (left), AG-14 (middle), and Cpd 17b (right) with the FPR1 binding pocket, respectively. The residues of FPR1 within 4.5 Å to the atoms of the ligands are shown as green licorice.

In functional studies, peptides without the N-formyl group have been shown to be much less potent in the activation of FPR1^26^. To illustrate the structural basis for formyl peptide recognition, docking analysis was conducted with MLF (Supplementary Data 1), the non-formyl sibling of fMLF (Fig. 4a). This ligand lacks contact with the critical residues R201^5.38^ and R205^5.42^ despite hydrophobic interactions with multiple contacts in the binding pocket (Fig. 4b, c). This finding is consistent with an early report that MLF was 3 orders of magnitude less potent in FPR1 binding^26^. tBoc-MLF, with 3 methyl groups at its N-terminus (Fig. 4a, Supplementary Data 2), is an antagonist of FPR1^26^. Docking analysis revealed a different pose for interaction with the FPR1 binding pocket (Fig. 4b, c). This ligand can possibly form hydrogen bond between the backbone carbonyl group of its Met and Y257^6.51^ of FPR1. The carboxyl group of Phe (F3) is located very close to R201^5.38^, but without any polar interaction. There is no observed contact between tBoc-MLF and critical residues in the binding pocket including R201^5.38^ and R205^5.42^, which may explain the pharmacological properties of tBoc-MLF as a FPR1 antagonist. Taken together, both ligands failed to properly insert into the FPR1 binding pocket for contacts with key residues including D106^3.33^, R201^5.38^ and R205^5.42^.

FPR1 was able to bind a variety of ligands including non-formyl peptides and non-peptide small molecules^4^. Among these, WKYMVm is a highly potent peptide ligand of FPR1 (Kd = 3.9 nM) and FPR2 (Kd = 0.8 nM)^20^. Identified through random screening of a peptide library^27^, WKYMVm does not have an N-formyl group but contains a D-Met at its carboxyl terminus (Fig. 4d). Using the solved FPR1-Gi complex as template, we performed molecular docking to determine the mode of WKYMVm binding (Supplementary Data 3). Unlike fMLF and fMIFL, WKYMVm assumes a pose with its C-terminus inserted into the FPR1 binding pocket (Fig. 4e). This insertion mode is also observed in the structures of WKYMVm bound to FPR2 (PDB ID: 6LW5, 6OMM). It is predicted that the C-terminal D-Met interacts with R205^5.42^ to form hydrogen bond (Fig. 4f). When the D-Met was replaced with L-Met, the interaction with R205^5.42^ is weakened due to chirality of the amino acids, such that WKYMVM was ~20-fold less potent than WKYMVm in functional assays^28^. The docking model (Fig. 4f) also predicts that R201^5.38^ interacts with WKYMVm directly by forming a hydrogen bond with the carbonyl group of Met (M4). WKYMVm in the FPR1 ligand binding pocket is surrounded by F102^3.29^, L109^3.36^, F178^ECL2^, and T265^6.59^. Of note, T265^6.59^ but not F102^3.29^ also plays a role in its interaction with fMIFL (Table 1). Our docking model also predicts that the amide group of D-Met oscillates between D106^3.33^ and R205^5.42^. As mentioned above, there is a possibility of hydrogen bond formation between D106^3.33^ and the nitrogen atom in the amide group of D-Met (M6 in WKYMVm), thus providing additional stabilization to WKYMVm binding. The WKYMVm-bound FPR2 structure has been resolved and used here for comparison^22,23^. The solved structure of FPR2 with bound WKYMVm identified D281^7.32^ and E89^2.68^ for salt bridge formation with W1 and K2 in WKYMVm, respectively;^23^ however, these polar interactions were absent from FPR1 (Fig. 4f), which may explain why WKYMVm is a less potent agonist for FPR1 than FPR2^29^.

**Table. 1:** cAMP responses of WT FPR1 and its mutants to selected agonists

Ligand	FPR1 construct	pEC_50_^#^, mean ± SEM	Ligand	FPR1 construct	pEC_50_^#^, mean ± SEM
**fMLF**	WT	9.1 ± 0.3	**WKYMVm**	WT	8.4 ± 0.2
	F81A	8.1 ± 0.4		F102A	7.9 ± 0.2
	F102A	8.5 ± 0.3		L109A	7.1 ± 0.3
	D106A	ND		F178A	7.5 ± 0.3
	D106N	ND		R201A	7.2 ± 0.2
	L109A	8.1 ± 0.2		R205A	7.7 ± 0.5
	F110A	8.6 ± 0.5		T265A	7.3 ± 0.2
	R201A	ND	**AG-14**	WT	6.4 ± 0.2
	R205A	ND		R84A	5.9 ± 0.2
	R201A-R205A	ND		R201A	5.8 ± 0.3
				R205A	6.0 ± 0.3
	T265A	7.8 ± 0.3		W254A	5.3 ± 0.2
	V283A	8.8 ± 0.2		Y257A	5.6 ± 0.2
	F291A	8.4 ± 0.2		F291A	5.7 ± 0.1
**fMIFL**	WT	10.2 ± 0.3	**Compound 17b**	WT	6.0 ± 0.2
	D106A	ND		R201A	6.0 ± 0.2
	D106N	ND		R205A	5.6 ± 0.3
	R201A	8.2 ± 0.3		W254A	4.7 ± 0.3
	R205A	8.6 ± 0.4		T265A	5.3 ± 0.2
	R201A-R205A	ND		F291A	5.3 ± 0.2

# The pEC50 values were calculated from the dose-response curves of FPR1 and its mutants in inhibiting forskolin-elevated cAMP concentrations. ND (not determined) refers to data where the pEC50 values were not accurate due to weak responses (cAMP inhibition < 30%). Data are obtained from three independent experiments, each in duplicates.

In addition to peptide ligands, FPR1 and FPR2 bind ligands of other compositions^17^. Multiple small molecules have been identified for both receptors through high-throughput screening^30,31^. AG-14 (Fig. 4d) is one of a series of small molecule agonists for FPR1^31^. When placed in the FPR1 binding pocket through molecular docking, AG-14 exhibits multiple contacts including formation of hydrogen bonds with R205^5.42^ and R84^2.63^ (Fig. 4e, f, Supplementary Data 4). There are also hydrophobic interactions between AG-14 and residues in the FPR1 binding pocket, including R201^5.38^, W254^6.48^, Y257^6.51^ and F291^7.43^. Another small molecule termed Compound 17b (cpd17b) is a ligand of both FPR1 and FPR2^32^ with cardiac protective property in studies using mice^33^ that express the mFpr1^34^. Cpd17b fits well into the FPR1-Gi complex model and may have multiple contacts with the FPR1 binding pocket (Fig. 4d–f, Supplementary Data 5).

### Functional analysis of FPR1-ligand interaction

Following FPR1 structural analysis, site-directed mutagenesis was conducted to determine the effects of alanine substitutions on selected amino acids in the FPR1 binding pocket. These included polar residues predicted to form hydrogen bonds with the ligands (D106^3.33^, R201^5.38^, and R205^5.42^), as well as non-polar residues that might contribute to the hydrophobic environment surrounding the ligands (Table 1). The mutagenized FPR1 was expressed by transfection and examined for cAMP concentration reduction following fMLF or fMIFL stimulation, an indication of Gαi activation.

**Fig. 5:**
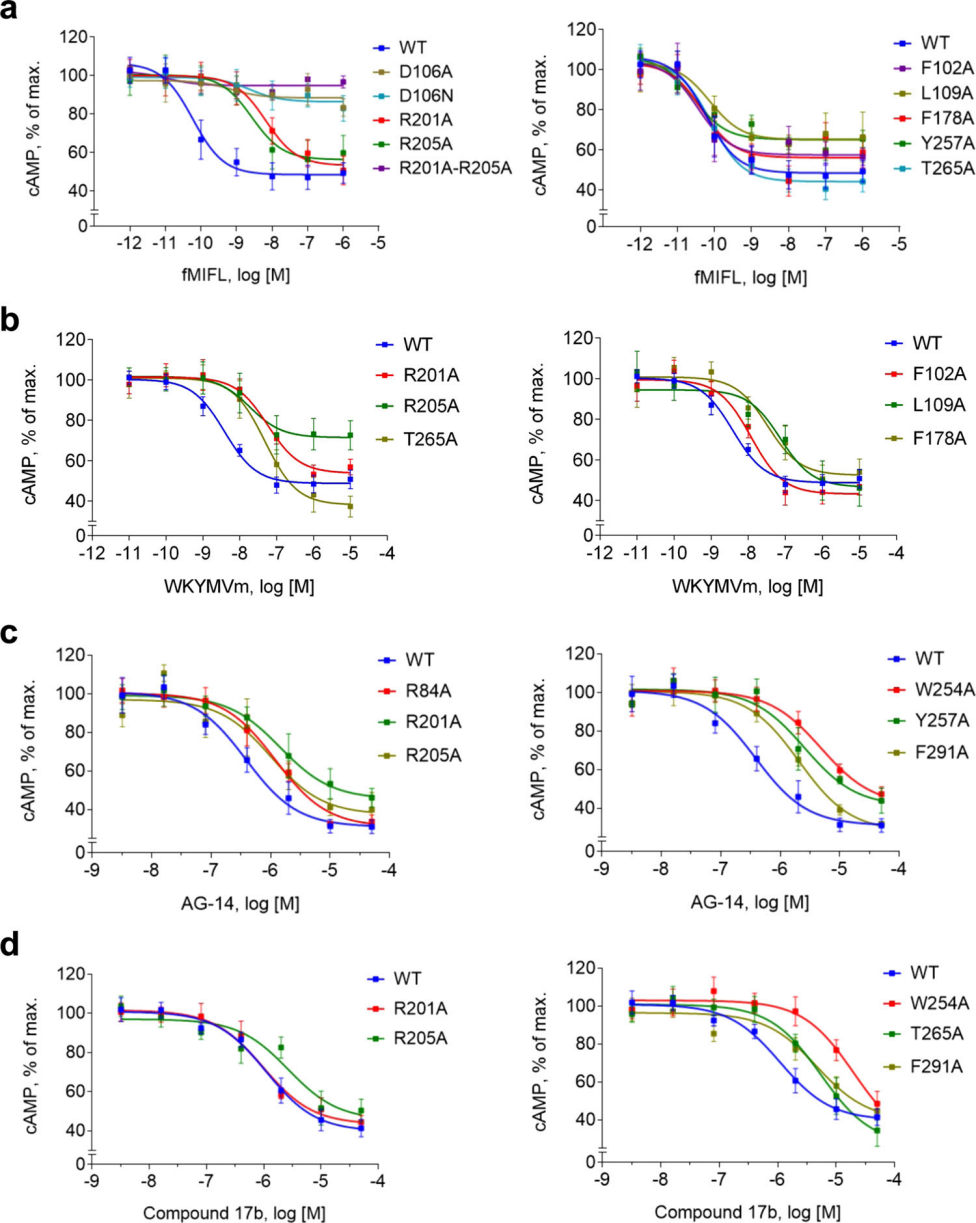
Dose-response curves of FPR1 and its mutants in cAMP inhibition assays. FPR1 and selected mutants were expressed in transiently transfected cells. The receptors were stimulated with different concentrations of the indicated agonists, fMIFL (**a**), WKYMVm (**b**), AG-14 (**c**) or Compound 17b (**d**) plus forskolin for 30 min. Changes in cytoplasmic cAMP concentrations were measured and data were plotted with the maximal cAMP concentrations set as 100%. Data are shown as mean ± SEM of three independent experiments, each in duplicates. Source data are provided as a Source Data file.

We first determined whether the FPR1 mutants were properly expressed on the cell surface using an anti-FPR1 mAb (see *Methods*). Alanine substitution at D106^3.33^, F110^3.37^ and R201^5.38^ + R205^5.42^ abrogated cell surface expression of the mutant receptors (Supplementary Fig. 8, upper panel). Since the antibody used was prepared against full FPR1 protein and therefore may be conformation-sensitive, we further determined whether these mutant FPR1 proteins could be expressed on the plasma membrane by introducing a FLAG tag to their N-termini. Flow cytometry analysis found that these mutant receptors were readily detectable by the M2 anti-FLAG antibody (Supplementary Fig. 8, lower panel). The findings suggest that alanine substitutions of these amino acids caused profound conformational changes in FPR1. Indeed, the D106A and R201A/R205A mutants failed to respond to fMIFL stimulation with Gαi activation as shown in cAMP inhibition assays (Fig. 5a). It is interesting to note that fMLF-bound receptor is more susceptible to single substitutions at R201^5.38^ or R205^5.42^ which abrogated the fMLF-induced response (Table 1). Therefore, peptide length has an impact on the binding to FPR1 as well as efficacy. The added amino acids in the carboxyl terminus of fMIFL mostly likely interact with the hydrophobic cap formed by R84^2,63^, F102^3.29^, F178^ECL2^ for further stabilization of the ligand in the FPR1 binding pocket. Whereas single substitutions of the charged residues produced significant effect in functional assays, single substitutions of the non-polar residues produced much less reduction in potency (Fig. 5a and Table 1) probably because the non-polar residues work together to form hydrophobic pockets surrounding the formyl peptide ligands.

WKYMVm does not contain an N-formy group but its carboxyl D-Met plays a role similar to that of fMet in formyl peptides. Functional studies confirmed this prediction, as replacing the D-Met with L-Met causes a ~20-fold reduction in potency. In cAMP inhibition assays, alanine substitution of R201^5.38^ or R205^5.42^ caused a significant right-shift of the dose-response curve (Fig. 5b). The hydrophobic clusters that surround Met4 is also important to the binding of WKYMVm, as substitution of L109^3.36^, F178^ECL2^ and T265^6.59^ produced similar reductions in potency (Fig. 5b). For AG-14, a much smaller molecule than WKYMVm that probably has to use all available contacts for FPR1 binding and receptor activation, alanine substitution at R201^5.38^, R205^5.42^, R84^2.63^, W254^6.48^, Y257^6.51^ and F291^7.43^ produced expected reduction in cAMP inhibition assays, with more obvious inhibition at W254^6.48^ and Y257^6.51^ (Fig. 5c). Consistent with our docking model, the potency of Cpd17b to stimulate the cAMP response decreased with the R205^5.42^A, T265^6.59^A, F291^7.43^A and particularly W254^6.48^A substitutions (Fig. 5d). Taken together, results from the functional assays support the proposed models based on the cryo-EM structure of FPR1.

### Structural basis for FPR1 coupling to Gi proteins

**Fig. 6:**
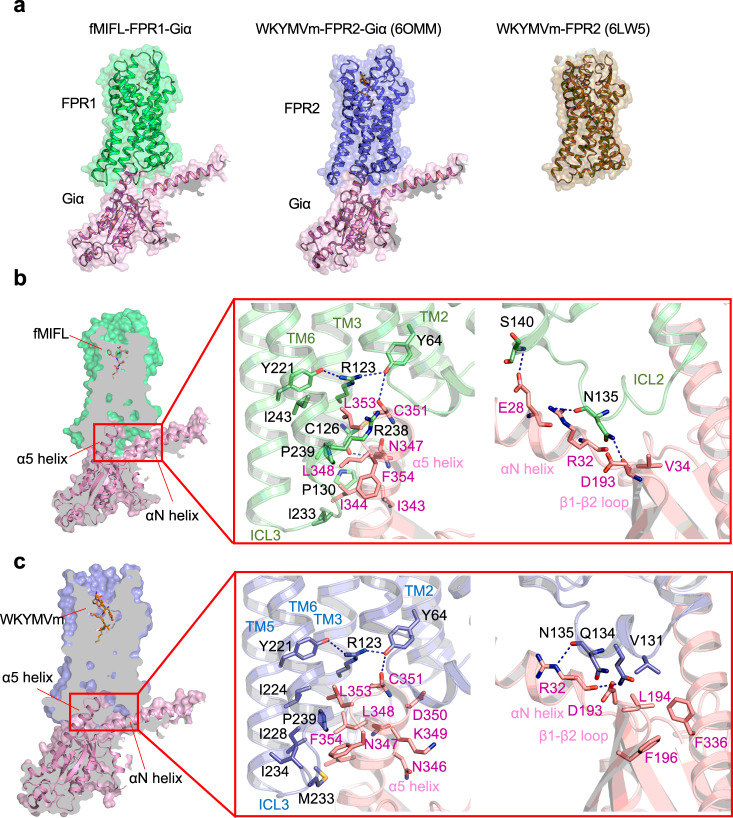
Interface of G_αi_ with FPR1 and FPR2. **a** Overview of the Gαi (pink) interacting with FPR1 (green, left) and FPR2 (blue, middle, PDB ID: 6OMM). The structures are front view from the intracellular side, showing in cartoon overlap with surface in 50% transparency. The crystal structure of FPR2 (right) is colored in olive and showed in cartoon and surface. **b** Slab view showing the interactions between FPR1 and Gαi protein (left). The region TM2, TM3, TM6, and ICL3 of FPR1 form direct contact with α5 helix of Gαi protein (middle). C126 in TM3, Y64 in TM2 forms hydrogen bond to N347, N351 in α5 helix of Gαi protein, respectively. ICL2 of FPR1 has polar interactions with αN helix and β1-β2 loop of Gαi protein (right). **c** Slab view showing the interactions between FPR2 and α5 helix of Gαi protein (left), as well as interactions of ICL2 of FPR2 with αN helix and β1-β2 loop of Gαi protein (right).

FPR1 is functionally coupled to Gi proteins as shown in early studies using pertussis toxin^12,13^. The structural features of FPR1 for Gi coupling was next investigated. The overall structures of the FPR1-Gi complex bound to fMIFL and the FPR2-Gi complex bound to WKYMVm indicate a similar Gi-protein coupling mode (Fig. 6a). The main interface is composed of the cavity formed by TM2, TM3, and TM6 with the C-terminal α5 helix of Gαi. In the structure of the FPR1-Gi complex (Fig. 6b), the C-terminus of Gαi penetrates the cavity at the cytoplasmic region of FPR1. The polar residues D193, N347, and D315 of Gαi formed hydrogen bonds with N135^ICL2^, C126^3.53^, and K235^ICL3^, respectively (Fig. 6b). Such a polar interaction network is similar to that in the FPR2-Gαi interface (Fig. 6c), which was formed by C351, R24, D193 of Gαi with Y64^2.43^, D134^ICL2^, and N135^ICL2^ of FPR2^22^. The hydrophobic residues I343, I344, L348, and L353 at the α5 helix of Gαi were surrounded by P130^ICL2^, V127^3.54^, L243^6.37^, P239^6.33^, and L233^ICL3^ of FPR1 (Fig. 6b), forming a hydrophobic cluster. Besides the TM domains, the cytoplasmic loops involved ICL2 and ICL3 are found to contact with αN helix and αN-β1 loop of Gαi protein. A hydrophobic cluster of FPR1-Gi interface is formed near the αN of Gαi containing E28, R32, and V34, interacting with Q134^ICL2^ and T138^ICL2^ of FPR1 (Fig. 6c). Although the ICL2 and ICL3 regions of FPR1 and FPR2 are highly conserved, there are some differences in the interactions of Gαi with FPR1 and FPR2. In the polar interaction network, major polar interaction is formed by ICL2 in FPR2 (Fig. 6c), while K235^ICL3^ can form a polar interaction with Gαi besides ICL2.

## Discussion

Here we investigated the structural basis for receptor recognition of peptides with N-formyl methionine, which is a hallmark of bacterial and mitochondrial protein synthesis. N-formyl peptides of various sequences have been identified from bacteria, and the cognate receptors for these peptide ligands have been found in phagocytes of humans and other mammals^35^. Human neutrophils respond to sub-nanomolar concentrations of N-formyl peptides such as fMLF, whereas mouse neutrophils are less efficient in fMLF recognition but are highly responsive to longer formyl peptides such as fMIFL and fMIVIL^21^. Genetically altered mice lacking *Fpr1* are more susceptible to Listeria infection with a higher mortality rate^36^. Likewise, recognition of N-formyl peptides from mitochondria plays a role in clearance of cell debris and restoration of tissues homeostasis^6,9,14^.

In the present study, the structures of human FPR1-Gi complex bound to two formyl peptides were obtained by cryo-EM at global resolutions of 2.8 Å (for fMIFL) and 2.9 Å (for fMLF). Analysis of the structure identified the R201^5.38^XXXR205^5.42^ motif that is present only in GPCRs known to bind formyl peptides, namely human FPR1 and FPR2, and mouse Fpr1. Of the two Arg residues in this motif, R205^5.42^ has been suggested previously to interact with the N-formyl group in fMLF and a similar ligand used for photoaffinity cross-linking^37^. While our structural and mutagenic studies have indeed identified an important role for R205^5.42^ in formyl peptide recognition, it is R201^5.38^ that directly contacts the N-formyl group in fMLF and fMIFL. Cryo-EM structures of the FPR1-Gi complex bound to fMLF and fMIFL show that the N-formyl group and the nitrogen atom on R201^5.38^ are very close to each other for hydrogen bond formation (Fig. 2c–f, Supplementary Fig. 5c-f). Consistent with the structural analysis, Ala substitution of R201^5.38^ markedly compromised recognition of fMLF and fMIFL (Fig. 5a; Table 1). While R201^5.38^ is unique to FPR1 and FPR2, R205^5.42^ is present in a few GPCRs that share sequence homology with FPR1 (Supplementary Fig. 9): In the C5a receptor (C5aR) as R206^5.42^ and the chemerin receptor 1 (chemokine-like receptor 1, CMKLR1) as R224^5.42^. In C5aR, R206^5.42^ is required for high affinity binding and interaction with the carboxyl R74 of the C5a ligand^38^. The functional role for R224^5.42^ in CMKLR1 has not been reported. The fact that both of these receptors lack R201^5.38^ as well as the ability to bind formyl peptides strongly indicates that R205^5.42^ alone is insufficient for recognition of N-formyl peptides. It is predicted that these Arg work together in the context of the R201^5.38^XXXR205^5.42^ motif for binding of formyl peptide and activation of FPR1.

D106^3.33^, like R201^5.38^, is found only in formyl peptide-binding receptors. Based on our cryo-EM structure of FPR1, D106^3.33^ is in close contact with fMet1, and either of its OD1 (oxygen atom without hydrogen) or OD2 (oxygen atom with hydrogen) may form hydrogen bond with the nitrogen atom on fMet1. This result was confirmed by MD simulations (Fig. 3). Ala or Asn substitution of D106^3.33^ abrogated formyl peptide-induced cAMP inhibition (Fig. 5) despite cell surface expression of the receptor. However, the mutant receptors were not recognized by an mAb produced against whole FPR1 exogenously expressed in a cell line, suggesting alteration of the overall structure of FPR1. MD simulation of the FPR1 structure identified a salt bridge between D106^3.33^ and R201^5.38^ (Fig. 3), with a possible role in maintaining the FPR1 structure in unbound state. However, single substitution of R201 with Ala did not produce the same overall structural change to FPR1, as the R201A mutant was readily detectible by the conformation-sensitive mAb and was able to bind fMIFL with lower affinity. Dual substitution (R201A/R205A) produced the same effect on FPR1 detection by the mAb as D106A did, and the double-mutant completely lost functionality in cAMP inhibition assays (Fig. 5a). A likely explanation is that R201^5.38^ and R205^5.42^ may be functionally switchable in binding fMIFL and in the maintenance of the overall structure of FPR1 with possible salt bridge formation between D106^3.33^ and either R201^5.38^ or R205^5.42^.

MD simulation further support our cryo-EM structure of FPR1, including a hydrogen bond network in the bottom half of the binding pocket that involves the formyl oxygen, the oxygen of the carbonyl group on fMet1, the nitrogen atoms of fMet1 and Ile2, and amino acid residues of the binding pocket including D106^3.33^, R201^5.38^ and R205^5.42^ (summarized in Table 2). The interactions can be dynamic for optimal binding affinity. For example, D106^3.33^ may form hydrogen bonds through its OD1 and OD2 with multiple side chains of amino acids aligning the binding pocket. In addition to hydrogen bonds, electrostatic force involving negatively charged oxygens and positively changed nitrogens may play a role in formyl peptide binding and further stabilize formyl peptide binding to FPR1. At the extracellular tip of helix II, there are two positively charged residues R84^2.63^ and K85^2.64^, that were thought to play important roles for FPR1 binding in previous studies employing amino acid substitutions^18,39^. Based on our cryo-EM structure of FPR1, these two positively charged residues do not directly contact fMLF but form charge interactions with Phe3 of fMLF. In FPR2, the amino acids at these positions are S84^2.63^ and M85^2.64^, and the loss of the positive charges is attributable to the much lower affinity of fMLF to FPR2 (Kd = 105 nM) compared with FPR1 (Kd = 1.6 nM)^39^. In addition to hydrogen bonds and electrostatic interactions, hydrophobic interactions play an important role in formyl peptide ligand binding to FPR1. There are several clusters of hydrophobic pockets surrounding Leu2 and Phe3 of fMLF (Fig. 2 and Table 2), that appear in the upper half of the binding pocket and serve to stabilize the carboxyl portion of the formyl peptide ligands. In this regard, longer peptides benefit with more hydrophobic interactions, along with improved binding affinity. Our results show differences between a tripeptide (fMLF) and a tetrapeptide (fMIFL) in their reliance on hydrogen bond formation with R201^5.38^ and R205^5.42^, such that single Ala substitution of these arginines only caused a right shift of the fMIFL dose-response curves (Fig. 5) but abolished the functions of fMLF-bound mutant receptors (Table 1). One possible explanation is the hydrophobic cap formed with R84^2.63^, F102^3.29^ and F178^ECL2^, that interacts with fMIFL but not fMLF (Fig. 2; Table 2).

**Table 2:** Summary of predicted interactions between selected ligands and amino acid residues of the FPR1 receptor within 4.5Å

Ligands	Functional groups	Interacting residues on FPR1
fMIFL	N-Formyl group (CHO)	Hydrogen bonding between formyl oxygen and R201^5.38^.
	Methionine (M1).	Carbonyl oxygen of methionine (M1) forms hydrogen bonding with R205^5.42^.
		Possible to form hydrogen bond between amide nitrogen of methionine (M1) and D106^3.33^.
		Methionine (M1) is surrounded by a hydrophobic pocket formed by L109^3.36^, F110^3.37^, V113^3.40^, W254^6.48^ and Q258^6.52^.
	Isoleucine (I2).	Nitrogen atom of isoleucine (I2) may form hydrogen bond with D106^3.33^.
		Isoleucine (I2) is surrounded by a hydrophobic environment formed by F81^2.60^, V105^3.32^, F291^7.43^.
	Phenylalanine (F3).	The arene ring of phenylalanine (F3) forms hydrophobic interaction with T265^6.59^.
	Leucine (L4).	Leucine (L4) is surrounded by a hydrophobic cap formed by R84^2.63^, F102^3.29^, F178^ECL2^.
fMLF	N-Formyl group (CHO).	Hydrogen bonding between formyl oxygen and R201^5.38^.
	Methionine (M1).	Carbonyl oxygen of methionine (M1) forms hydrogen bonding with R205^5.42^.
		Possible to form hydrogen bond between amide nitrogen of methionine (M1) and D106^3.33^.
	Leucine (L2).	Nitrogen atom of leucine (L2) may form hydrogen bond with D106^3.33^.
		Leucine (L2) is surrounded by a hydrophobic pocket formed by F81^2.60^, V105^3.32^ and L109^3.36^.
	Phenylalanine (F3).	Hydrophobic interaction with F102^3.29^, T265^6.59^, and I268^ECL3^.
WKYMVm	D-Met (m6).	The C-terminal D-Met (m6) interacts with R205^5.42^ to form hydrogen bond. The amide group of D-Met oscillates between D106^3.33^ and R205^5.42^.
	The overall ligand.	WKYMVm is surrounded by F102^3.29^, L109^3.36^, F178^ECL2^, and T265^6.59^.
AG-14	The overall ligand.	Multiple contacts including formation of hydrogen bonds with R205^5.42^ and R84^2.63^.Hydrophobic interactions between AG-14 and R201^5.38^, W254^6.48^, Y257^6.51^ and F291^7.43^.
Cpd17b	The overall ligand.	Multiple contacts with the FPR1 binding pocket, including D106^3.33^, R201^5.38^ and R205^5.42^.

Our cryo-EM structure of FPR1 showed that the N-formyl Met insert into the bottom of the FPR1 binding pocket, allowing maximal contact of the fMet with D106^3.33^, R201^5.38^ and R205^5.42^ for hydrogen bond formation. In contrast, MLF, the non-formyl sibling of fMLF, was not properly oriented in the FPR1 binding pocket (Fig. 4). Likewise, the FPR1 antagonist tBOC-MLF was not able to insert its N-terminus deep into the binding pocket, suggesting that the N-formyl group may guide the ligand for proper positioning in the FPR1 binding pocket. It is notable that some synthetic peptides without an N-formyl group can also serve as potent agonists for FPR1. WKYMVm, a synthetic hexapeptide selected for potency from high-throughput screening of a peptide library, was analyzed in this study and was found to utilize both R201^5.38^ and R205^5.42^ for optimal agonism (pEC_50_ = 8.4 ± 0.2). WKYMVm also interacts with D106^3.33^ through the carboxyl D-Met that plays a role similar to fMet in formyl peptides. Likewise, AG-14, a small molecule agonist of FPR1 identified by high-throughput screening, was found to form contacts with R205^5.42^ and R84^2.63^. AG-14 (M.W. 404.42) is much smaller than WKYMVm (M.W. 856.11) and therefore forms fewer contacts with the FPR1 binding pocket, resulting in low potency (pEC_50_ = 6.4 ± 0.2 for cAMP inhibition). Taken together, these results indicate that FPR1 selectivity is determined by multiple factors including proper orientation during ligand entry (e.g., fMLF vs. MLF), contacts with residues at the bottom of the binding pocket (e.g., fMLF vs. tBOC-MLF, and Ala substitutions of D106^3.33^, R201^5.38^ and R205^5.42^). Hydrophobic interactions and electrostatic forces also play important roles in ligand selectivity of FPR1 and FPR2 (e.g., fMLF binding in FPR1 vs. FPR2, and fMLF vs. fMIFL in susceptibility to single substitutions at R201^5.38^ and R205^5.42^). For non-formyl peptides (e.g., WKYMVm) and non-peptide ligands (e.g., AG-14), FPR1 selectivity is determined by ligand occupancy and contacts with the binding pocket, in addition to interactions with key residues such as R201^5.38^, R205^5.42^ and R84^2.63^.

FPR2 is a homolog of FPR1 with 69% identical amino acids^4^. Like FPR1, it contains the R201^5.38^XXXR205^5.42^ motif and binds N-formyl peptides. Unlike FPR1, the entrance of the FPR2 binding pocket is wider^22,23^ and can accommodate more diverse ligands. When comparing the structures of FPR1 with FPR2, it is found that the extracellular side of TM5, ECL2, and ECL3 in FPR1 show an inward movement, leading a narrower mouth of FPR1 (Supplementary Fig. 10a). The electrostatic interactions formed by positively charged residues such as R84^2.63^ and K85^2.64^ in FPR1 are absent from FPR2 (Supplementary Fig. 10b), as the opening of the ligand binding pocket in FPR2 is lined with negatively charged residues of E89^ECL1^ and D281^7.32^. In FPR1, R84^2.63^ can form polar interaction with D284^7.36^. A comparison of fMIFL-bound FPR1 with that of FPR2 from the same views (Supplementary Fig. 10c, d) found many similar features including interactions with D106^3.33^, R201^5.38^, and R205^5.42^ of FPR2. However, the charge environment formation in the binding cavity of FPR1 and FPR2 are different^22^. With respect to formyl peptide binding, FPR2 displays much lower affinities (Kd in 100–500 nM) for short peptides such as fMLF^20^. Molecular docking analysis have shown that, without a hydrophobic cap at the opening of the binding pocket, the tripeptide does not fit snuggly in FPR2 as it does in FPR1^22^.

Studies of formyl peptide receptors continue to draw interests mainly because of its role in inflammation and resolution, and its intriguing features of binding diverse ligands. While this work was under revision, two laboratories published their findings of the FPR1-Gαi structures complexed with formyl peptides of different lengths^40,41^. More detailed analyses of features common and distinct between FPR1 and FPR2 were also provided in these studies. Although the lengths and composition of the formyl peptides used in these independent studies were different from tripeptide to nonapeptide, there are common features including the pose of formyl peptides in the binding pocket, the critical roles for D106^3.33^, R201^5.38^ and R205^5.42^ in their interactions with fMet and the formyl group, and the hydrophobic environment surrounding some residues of the formyl peptide ligands. Targeting these features may be a strategy for intervention of formyl peptide binding and development of novel therapies.

## Methods

### Construction, expression and purification of FPR1

*Homo sapiens* FPR1 cDNA (Gene ID: 2357) was cloned into pFastBac1 vector (Invitrogen, Carlsbad, CA, USA) with an N-terminal FLAG tag and a C-terminal 6 × His tag. The construct was transformed into *E. coli* (DH10Bac, Invitrogen) to obtain the recombinant bacmid. The recombinant baculovirus was prepared in *Spodoptera frugiperda* (Sf9) insect cells using the Bac-to-Bac system (Invitrogen). Sf9 cells were grown to a density of 4 million per ml and infected with the baculovirus at a ratio of 1:40. Cells were collected after 48 h and stored at −80 °C.

For FPR1 purification, a total of 3 L frozen cell pellets were lysed in 150 mL lysis buffer containing 10 mM hydroxyethyl piperazine ethanesulfonic acid (HEPES) (pH 7.5), 1 mM ethylenediamine tetra-acetic acid (EDTA), 1 mg/ml iodoacetamide, 2.5 μg/ml leupeptin, and 0.16 mg/ml benzamidine. Cell membranes were collected by centrifugation and solubilized in 100 mL solubilization buffer containing 20 mM HEPES (pH 7.5), 100 mM NaCl, 1% dodecyl maltoside (DDM), 0.1% cholesteryl hemisuccinate (CHS), 10% glycerol, 1 mg/ml iodoacetamide, 2.5 μg/ml leupeptin, and 0.16 mg/ml benzamidine. After centrifugation to remove the insoluble debris, the supernatant was supplemented with 2 mM CaCl_2_ and loaded onto 3 mL anti-FLAG M1 affinity resin. The resin was extensively washed, and the detergent exchanged from DDM to 0.01% lauryl maltose neopentyl glycol (LMNG) during wash steps. Proteins were eluted with 20 mM HEPES pH 7.5, 100 mM NaCl, 0.01% LMNG, 0.001% CHS, 200 μM FLAG peptide, and 5 mM EDTA. The elution was concentrated and loaded onto Superdex® 200 Increase 10/300 size exclusion column (GE Healthcare Life Sciences, Chicago, IL, USA) with a running buffer of 20 mM HEPES (pH 7.5), 100 mM NaCl, 0.01% LMNG and 0.001% CHS. The peak fractions (Supplementary Fig. 1) were collected and concentrated, fast frozen in liquid nitrogen (LN_2_) and stored at −80 °C until use.

### Construction, expression and purification of G_i1_ heterotrimer and scFv16

For G_i1_ heterotrimer expression, Human Gα_i_ cDNA was cloned into pFastbac1 vector, and N-terminal 6 × His-tagged human Gβ_1_ and non-tagged Gγ_2_ were cloned into pFastBac-Dual vector (Invitrogen). The baculovirus was prepared in the same way as FPR1. *Trichoplusia ni* Hi5 insect cells (Invitrogen) were grown to a density of 2.5 million per ml and infected with the above Gα_i_ and Gβγ baculoviruses at a ratio of 1:40 and 1:400, respectively. Cells were collected after 48 h and stored at −80 °C.

For purification of G_i1_ heterotrimer, cells were lysed in 10 mM HEPES (pH 7.5) supplemented with 10 μM guanosine 5′-diphosphate (GDP) sodium salt and 1 mM MgCl_2_. Cell membranes were collected and solubilized in 1% sodium cholate and 0.05% DDM supplemented with 25 μM GDP and 1 mM MgCl_2_. After solubilization, the supernatant was collected and loaded onto Ni-NTA resin column. The resin was extensively washed, and the detergent was exchanged to 0.08% DDM during wash step. G_i1_ heterotrimer was eluted with 20 mM HEPES (pH 7.5), 100 mM NaCl, 0.08% DDM, 250 mM imidazole, 100 μM tris(2-carboxyethyl) phosphine (TCEP), 25 μM GDP and 1 mM MgCl_2_. After elution, 1 μl lambda phosphatase (New England Biolabs, Ipswich, MA, USA), 1 μl calf intestinal alkaline phosphatase (CIP) (New England Biolabs) and 1 mM MnCl_2_ was added, and the mixture was incubated on ice overnight. The next day, the protein was concentrated to about 20 mg/ml, fast frozen in LN_2_ and stored at −80 °C.

The antibody fragment scFv16 was purified as a secretory protein, using baculovirus in the same way as FPR1. *Trichoplusia ni* Hi5 insect cells were grown to a density of 2.5 million per ml and infected with the virus at a ratio of 1:40. After 60 h; the supernatant was collected and loaded onto Ni-NTA resin column. The column was washed with 20 mM HEPES (pH 7.5), 500 mM NaCl and protein eluted by 20 mM HEPES (pH 7.5), 500 mM NaCl and 250 mM imidazole. The eluted proteins were concentrated and loaded onto Superdex 200 increase 10/300 size exclusion column (GE Healthcare). The peak fractions were collected and concentrated, fast frozen in LN_2_ and stored at −80 °C.

### FPR1-G_i1_-scFv16 complex formation and purification

For complex formation, 0.4 mg purified FPR1 was incubated with 1 mg G_i1_ in a buffer of 20 mM HEPES (pH 7.5), 100 mM NaCl, 1% LMNG, 100 µM fMLF (or fMIFL) on ice for 2 h. Then apyrase with 10 mM MgCl_2_ was added to remove GDP from the system and the mixture was incubated on ice overnight. The mixture was then diluted in a buffer of 20 mM HEPES (pH 7.5), 100 mM NaCl, 0.01% LMNG, 0.003% GDN, 0.001% CHS, 10 µM fMLF (or fMIFL) and loaded onto anti-FLAG M1 affinity resin column. The resin was extensively washed, and detergent concentration was decreased to 0.003% LMNG and 0.001% GDN during the wash steps. The complex was eluted with the 20 mM HEPES (pH 7.5), 100 mM NaCl, 0.003% LMNG, 0.001% GDN, 0.0004% CHS, 10 µM fMLF, 200 μM FLAG peptide, 5 mM EDTA and incubated with 0.25 mg purified scFv16 for 2 h on ice. Then the FPR1-G_i1_-scFv16 complex was loaded onto Superdex 200 Increase 10/300 size exclusion column (GE) with running buffer (20 mM HEPES pH 7.5, 100 mM NaCl, 0.003% LMNG, 0.001% GDN, 0.0004% CHS, 100 μM TCEP). The monomeric complex peak was collected and concentrated for electron microscopy.

### Cryo-EM grid preparation and data collection

For cryo-EM grid preparation, the purified fMIFL- or fMLF-activated FPR1-Gi-scFv16 complex was concentrated to ~6 mg/ml and was loaded onto a holey carbon grid (Quantifoil R1.2/1.3 Au 200), which was glow discharged using Pie Scientific Tergeo Plasma Cleaner at 15 w under air for 1 min. The grids were blotted for 3.5 s and flash-frozen with Vitrobot (Mark IV, Thermo Fisher Scientific, Waltham, MA, USA). For data collection, the 300 kV Titan Krios Gi3 equipped with Gatan K3 Summit detector and GIF Quantum energy filter (Thermo Fisher Scientific) was operated in the counted-Nanoprobe mode. The movie stacks with 50 frames were automatically collected using SerialEM 3.8 software at a nominal magnification of 105,000X, corresponding to a pixel size of 0.85 Å. For fMLF-FPR1-Gi-scFv16 complex, the defocus range were from −1.2 μm to −2.0 μm. Each movie stack was exposed for 2.5 s and the total dose was about 55 e^−^/Å^2^. For fMIFL-FPR1-Gi-scFv16 complex, the defocus range were from −1.0 μm to −1.8 μm and each movie stack was exposed for 3.75 s and the total dose was about 49.5 e^−^/Å^2^.

### Cryo-EM data processing

For fMLF-FPR1-Gi-scFv16 complex, a total number of 6508 movies were collected. Each movie stack was aligned using MotionCor2_1.3.0-Cuda101^42^. The Kai Zhang’s Gctf program (v. 1.06) was used to estimate the contrast transfer function (CTF)^43^. A total number of 4,310,681 particles were auto-picked using Laplacian-of-Gaussian filter in RELION 3.1^44^. Two rounds of reference-free 2D classification were performed with ten subsets, resulting a total number of 501,691 good particles. The 3D classification was performed with low-pass filtered μ-opioid receptor-G_i_ protein-scFv16 complex map (EMD-7868) as an initial model (Supplementary Fig. 2). Particles from two good classes were combined for the 3D auto-refinement, resulting a 3.6 Å resolution density map. Then the Bayesian polishing program was used to estimate trajectories of particle motion and the amount of cumulative beam damage, following with 3D auto-refinement. The map resolution was improved to 3.4 Å. The coordinates were exported to cryoSPARC 3.3.1 for a non-uniform refinement with a map with global resolution of 2.9 Å with FSC 0.143 criteria^45^. Local resolution was estimated using blocres implemented in cryoSPARC. Surface coloring of the density map was performed using UCSF Chimera 1.16^46^ (Supplementary Fig. 3).

For fMIFL-FPR1-Gi-scFv16 complex, all data processing steps were performed in RELION 3.1^44^. A total of 2,473 movies were collected, following with motion correction, CTF estimation and auto-mated particles picking as described above, resulting a total number of 1,690,578 particles. After one round of 2D classification, 575,272 particles were selected and subjected to the following 3D processing. Several rounds of 3D classification were conducted to exclude bad particles and resulting in a final subset of 230,890 good particles, which were then subjected to CTF refinement and Bayesian polishing. The coordinates were exported to cryoSPARC 3.3.1 for a non-uniform refinement, yielding a structure at 2.8 Å resolution. Local resolution was estimated in blocres and surface coloring of the density map was the same with the fMLF-FPR1-Gi-scFv16 complex.

### Model building and refinement

The homology model of FPR1 was generated by SWISS-MODEL using the activated FPR2 structure (from the structure of FPR2-Gi-scFv16, PDB: 6OMM) as template^22^. The model of Gi-scFv16 was taken from the structure of FPR2-Gi-scFv16. All models were docked into the electron density map using Phenix.dock_in_map^47^. The coordinates and geometry restrains of fMLF, fMIFL, and cholesterol were generated using Phenix.eLBOW^47^, and the ligands were manually fitted into the electron density map in Coot 0.9.7^48^. The starting model of fMLF-FPR1-Gi-scFv16 or fMIFL-FPR1-Gi-scFv16 was then subjected to iterative manual adjustment and real space refinement in Coot 0.9.7 and Phenix.Real_sapce_refinement^47^. The final refinement statistics were validated by MolProbity. To evaluate the potential model overfitting, the model was refined against the cryo-EM halfmap1 after all atoms were randomly displaced by 0.2 Å. FSC curves between the resulting model and the two half maps were calculated for cross-validation (Supplementary Fig. 3). The cryo-EM density map and model are shown for both formyl peptides, all seven transmembrane helices, helix 8, and α5 of Gα helices were shown in Supplementary Fig. 4.

### MD simulations of fMIFL-FPR1 and fMLF-FPR1 complexes

MD simulation was performed using GROMACS (version 2020.2)^49^. Protonation state of the FPR1 was assigned by the web server H^++^^50^ assuming pH 7.4, and CHARMM36m^51^ force field was employed in all simulations. Firstly, the system was energy minimized in 10,000 steps. Then 200 ns of restrained MD simulation was performed to fully relax and equilibrate the solvent and membrane structure at 303.1 K and 1.0 bar^52^, three independent 1-µs long production MD simulations were carried out for fMIFL-FPR1 and fMLF-FPR1 complexes, respectively. A total of 15,000 conformations were collected for each complex. Hydrogen bonds were identified based on cutoffs for the Donor-H⋯Acceptor (D–A) distance and angle. The criterion employed was angle > 120° and D–A distance < 2.5 Å in at least 10% of the trajectory. Representative hydrogen bond networks were characterized by minimizing the average D–A distance of all possible hydrogen bonds, and by maximizing the number of concurrent hydrogen bonds satisfying the criterion of D–A distance < 3 Å.

### Mutagenesis study

FPR1 cDNA in the pcDNA3.1(+) vector (Invitrogen) was used as a template for gene manipulation. The mutations of FPR1 were introduced in the receptor through overlap extension PCR with elaborately designed primers (GENEWIZ, Suzhou, China). The sequences of the primers were listed in Supplementary Table 2. Two fragments of FPR1 (separated at mutated positions) were assembled into pre-cut pcDNA3.1(+) vectors with the ClonExpress Ultra One Step Cloning Kit (Vazyme Biotech, C115). Plasmids with FPR1 mutations were confirmed by DNA sequencing (GENEWIZ).

Cell surface expression of FPR1 mutants was analyzed by flow cytometry. FPR1 and its mutants were transiently expressed in HeLa cells for 24 h. The cells were incubated on ice for 1 h with Alexa Fluor® 647-labeled anti-FPR1 antibodies (Becton Dickinson, Cat #565623; 1:50 diluted by HBSS buffer). The N-terminal FLAG-tagged WT and mutant receptors were detected with a FITC-labeled anti-FLAG antibody (M2; Sigma, Cat #F4049). After washing, cell fluorescence was detected by the Accuri C6 Plus flow cytometer (Becton Dickinson, Franklin Lakes, NJ, USA). Data were analyzed with Prism 6.0. Relative expression of FPR1 mutants was represented according to the fluorescence signals.

For functional studies, FPR1 and its mutants were expressed in HeLa cells as above. The cells were resuspended in HBSS buffer plus 5 mM HEPES, 0.1% BSA (w/v) and 0.5 mM 3-isobutyl-1-methylxanthine and seeded into 384-well plates. The tripeptide fMLF was provided by Sigma. WKYMVm and fMIFL were synthesized by ChinaPeptides (Shanghai, China). Compound 17b was synthesized by WuXi AppTec (Shanghai, China). AG-14 was synthesized by Sungening Biotechnology (Shenzhen, China). Different concentrations of the ligands were prepared plus a fixed dose of forskolin with the buffer above. The cells were stimulated by the ligands and 2.5 μM forskolin for 30 min in a cell incubator. Intracellular cAMP levels were measured with the LANCE Ultra cAMP kit (PerkinElmer, TRF0263) following the manufacturer’s instructions. In the measurements, signals of time resolved-fluorescence resonance energy transfer (TR-FRET) were detected by the EnVision 2105 multimode plate reader (PerkinElmer, Waltham, MA, USA). Intracellular cAMP levels were calculated according to the TR-FRET signals of the samples and cAMP standards.

### Molecular docking analysis of different ligands to FPR1

The cryo-EM structure of FPR1 was prepared for docking analysis, using the AutoDock Tool^53,54^. Hydrogen atoms were added to the receptor before running docking analysis edited in the Python Molecular Viewer (PMV, v 1.5.7). The 3D structures of fMLF, MLF, tBoc-MLF, WKYMVm, AG-14, and Compound 17b, fMIFL, were generated and optimized using Avogadro platform^55^. After ligands preparation, dockings of these ligands to FPR1 were performed with the AutoDock Tool^20^. The docking grid was centered on the centroid of fMLF. The docking parameters were performed with default settings and the Lamarckian genetic algorithm (LGA) was employed for docking process. Using the binding mode of fMLF in FPR1 in the cryo-EM structure as reference, the top-scoring conformations of the docking poses of every ligand were selected for clustering. After cluster analysis, the binding pose chosen from the optimal conformations was presented for the binding sites of the ligands and FPR1. In FPR2 docking analysis, the coordinates of FPR2 cryo-EM structure (PDB ID: 6OMM) are set as receptor. The parameters used in docking analysis of fMIFL to FPR2 is as same as that in FPR1 mentioned above. To verify the above method, the same docking parameters were applied to FPR1 with fMLF as the ligand, confirming a similar binding pose observed in the cryo-EM structure.

### Statistical analysis

The data were analyzed with Prism 6.0 (GraphPad, San Diego, CA). For dose-response analysis, the curves were plotted with the log[agonist] vs. response equation (three parameters) in the software. Data points were presented as the percentages (mean ± SEM) of the maximal cAMP level for each construct, from at least three independent experiments, as indicated in figure legends. The pEC_50_ values were the negative logarithm of the EC_50_ values, which were obtained from the dose-response curves. For cell surface expression, data points were presented as the percentages (mean ± SEM) of the flow cytometry fluorescence signals of WT FPR1. For statistical comparison, a *p*-value of 0.05 or lower is considered statistically significant.

### Reporting summary

Further information on research design is available in the Nature Research Reporting Summary linked to this article.

## Supplementary information

Supplementary Information

Description of Additional Supplementary Files

Supplementary Data 1

Supplementary Data 2

Supplementary Data 3

Supplementary Data 4

Supplementary Data 5

Reporting Summary

## Data Availability

All relevant data have been included in the manuscript and the Supplementary Information, except the following: The 3D cryo-EM density maps of the fMLF-FPR1-Gi-scFv16 and fMIFL-FPR1-Gi-scFv16 complex have been deposited in the Electron Microscopy Data Bank under the accession numbers EMD-31323 and EMD-31962, respectively. Atomic coordinates for the atomic model of fMLF-FPR1-Gi-scFv16 and fMIFL-FPR1-Gi-scFv16 have been deposited in the Protein Data Bank (PDB) under the accession numbers 7EUO and 7VFX, respectively. The structural models of WKYMVm-FPR2 are available in the PDB database under accession codes 6LW5 and 6OMM. The PDB file of the docking models of FPR1 to MLF, tBOC-MLF, WKYMVm, AG14, and compound 17b are provided as Supplementary Data 1–5, respectively. Source data are provided with this paper.

## Source data

Source Data
