# Supplementary material for: Structural basis for recognition of N-formyl peptides as pathogen-associated molecular patterns
Source: Nat Commun. 2022 Sep 5;13:5232. doi: 10.1038/s41467-022-32822-y (PMC9445081; doi:10.1038/s41467-022-32822-y)
Supplement: Supplementary file 1 — Supplementary Information [file 41467_2022_32822_MOESM1_ESM.pdf]

Supplementary Information

STRUCTURAL BASIS FOR RECOGNITION OF N-FORMYL PEPTIDES AS PATHOGEN-ASSOCIATED MOLECULAR PATTERNS

Geng Chen[1,8], Xiankun Wang[1,8], Qiwen Liao[1,8], Yunjun Ge[1,2,8], Haizhan Jiao[1,2], Qiang Chen[1], Yezhou Liu[1,3], Wenping Lyu[4], Lizhe Zhu[4], Gydo C. P. van Zundert[5], Michael J. Robertson[6], Georgios Skiniotis[6,7], Yang Du[1, ✉], Hongli Hu[1, ✉], Richard D. Ye[1,✉]

[1]Kobilka Institute of Innovative Drug Discovery, School of Medicine, The Chinese University of Hong Kong, Shenzhen, Guangdong 518172, China

[2]School of Life Sciences, University of Science and Technology of China, Anhui 230026, China

[3]Shenzhen Bay Laboratory, Shenzhen, Guangdong 518055, China

[4]Warshel Institute for Computational Biology, School of Medicine, The Chinese University of Hong Kong, Shenzhen, Guangdong 518172, China

[5]Schrödinger, New York, NY 10036, USA

[6]Department of Molecular and Cellular Physiology, Stanford University School of Medicine, Stanford, CA 94305, USA

[7]Department of Structural Biology, Stanford University School of Medicine, Stanford, CA 94305, USA

[8]These authors contributed equally: Geng Chen, Xiankun Wang, Qiwen Liao, Yunjun Ge

✉ yangdu@cuhk.edu.cn; honglihu@cuhk.edu.cn; richardye@cuhk.edu.cn

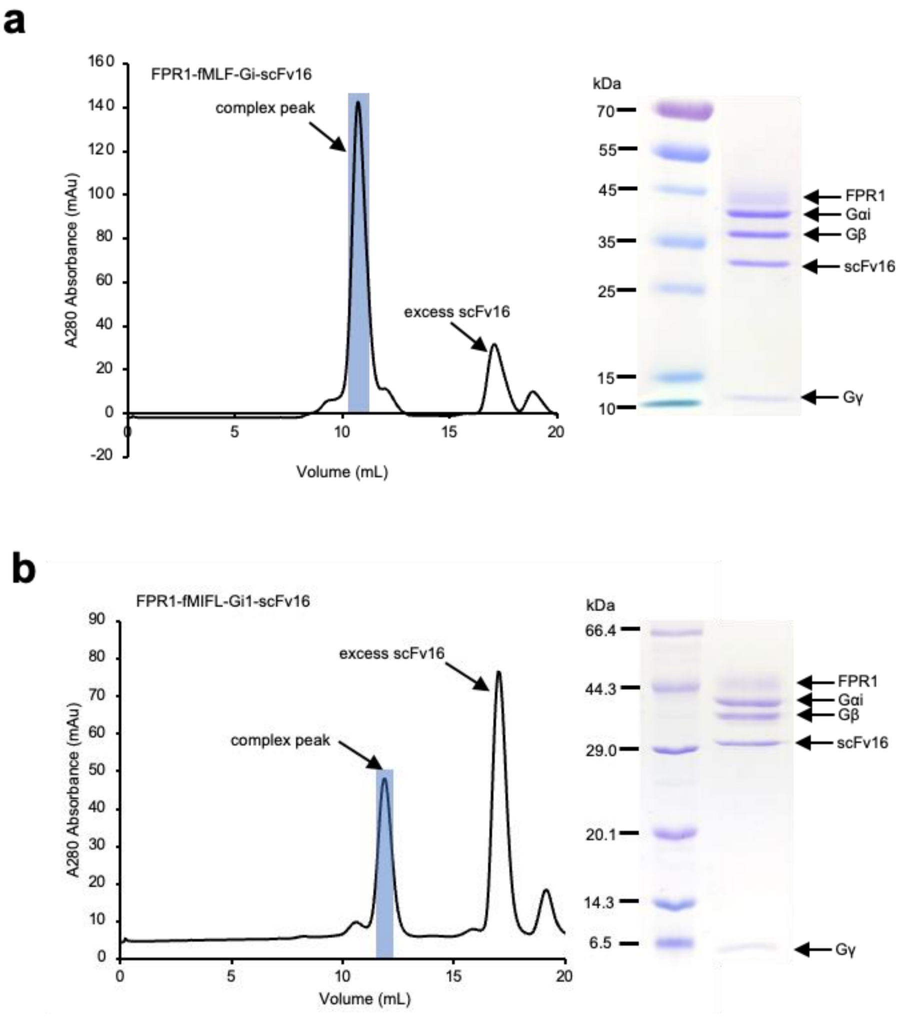

Supplementary Fig. 1. Purification of FPR1-Gi-scFv16 complexes bound to fMLF and fMIFL.

Shown are the collected peak fractions of FPR1-Gi-scFv16 complex bound to fMLF (**a**) and fMIFL (**b**). Size exclusion chromatography profile and SDS-PAGE staining image are from more than three independent experiments. See *Methods* for experimental details. Source data are provided as a Source Data file.

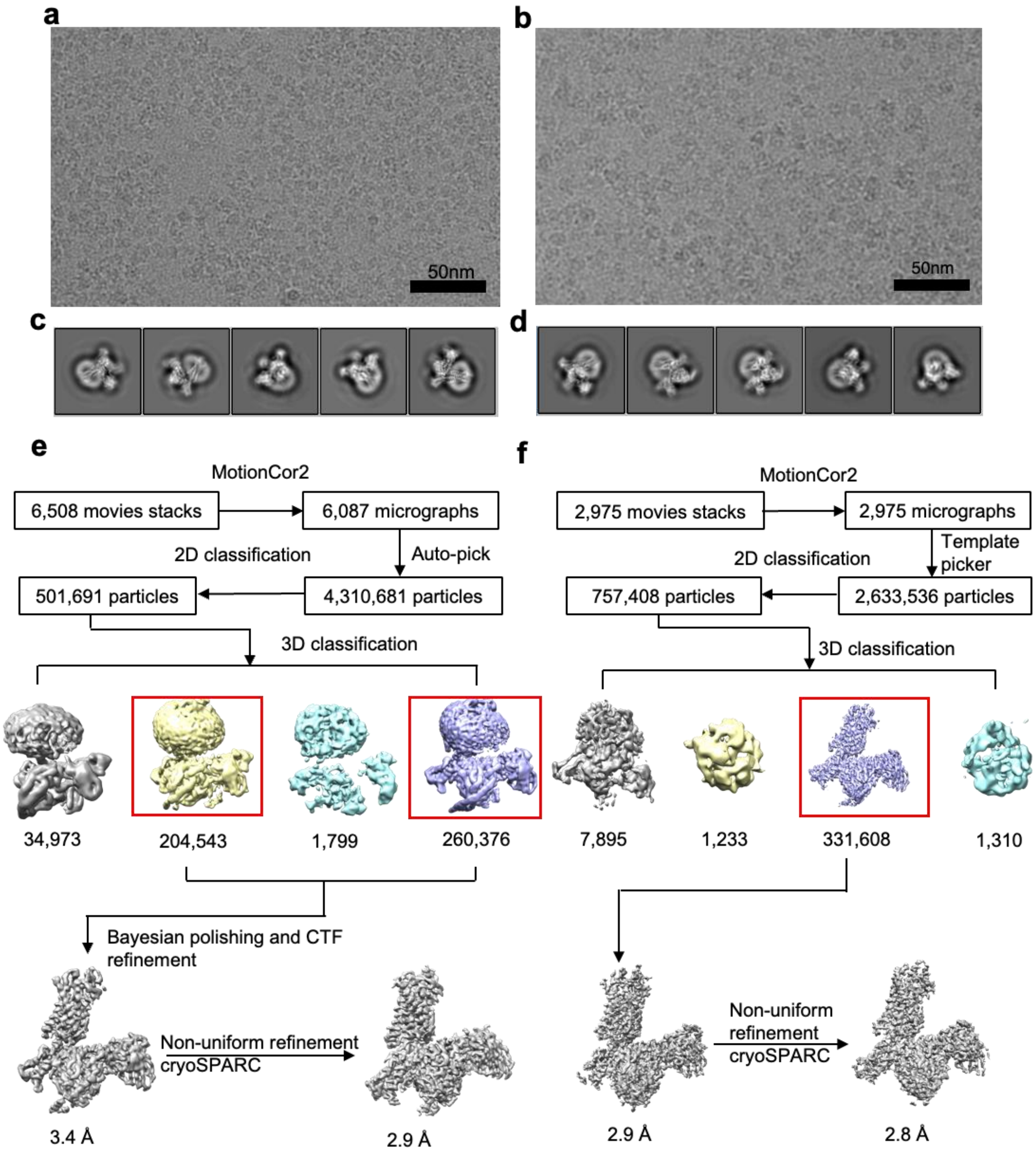

Supplementary Fig. 2. Cryo-EM data processing of FPR1- Gi-scFv16 bound to fMLF and fMIFL.

Representative raw cryo-EM image and 2D class averages of the receptor binding to fMLF from 6508 movies (**a, c**) and fMIFL from 2975 movies (**b, d**). Shown below the images is a cryo-EM data processing flow chart, including particle selection, classification, and 3D map reconstruction of the complex bound to fMLF (**e**) and fMIFL (**f**).

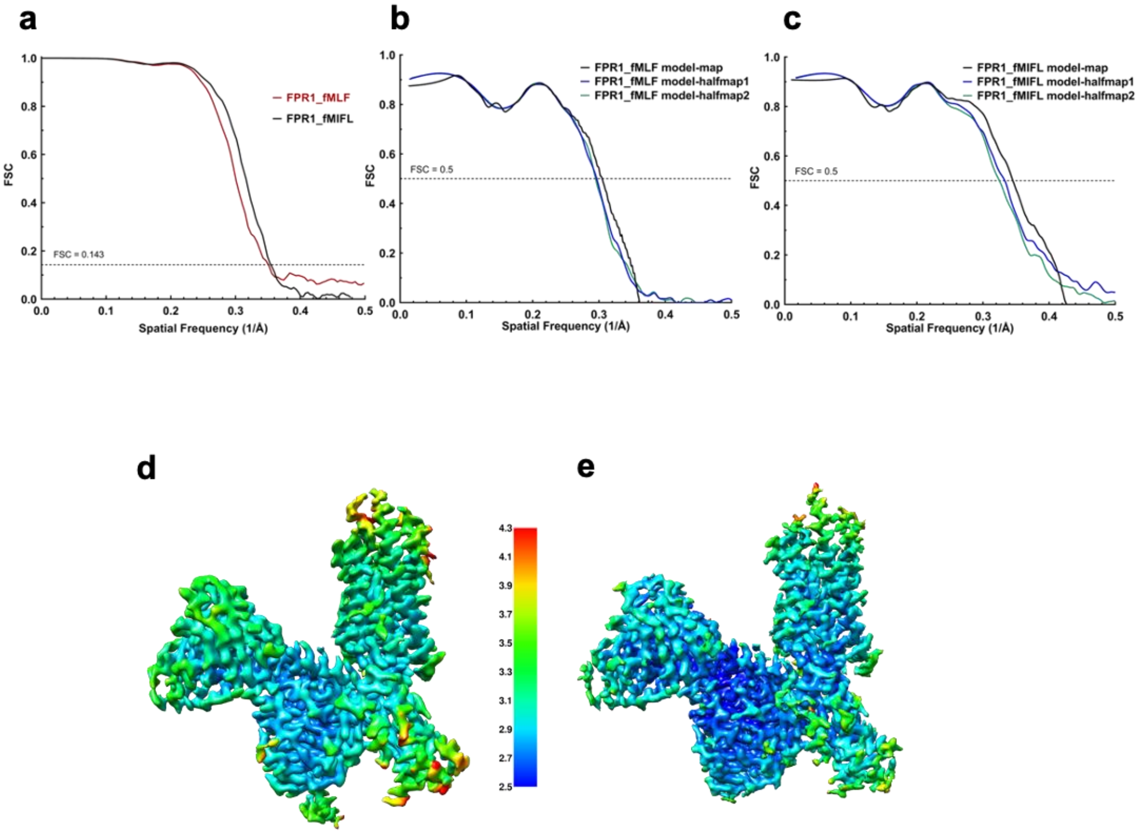

Supplementary Fig. 3. Map and Model Quality and Local Resolution.

a Fourier shell correlation (FSC) curves of two individual half maps indicating an average resolution of 2.84 Å (fMLF-FPR1-Gi-scFv16, Red) and 2.79 Å (fMIFL-FPR1-Gi-scFv16, Black) at 0.143 FSC. Model validations are shown for FPR1-Gi complexes activated by fMLF (**b**) and fMIFL (**c**), respectively. The FSC curves for model versus the EM map (black) and model versus two independent half maps (blue and green) were calculated with Phenix_M-triage. Analysis of the colored density map of fMLF-FPR1-Gi-scFv16 (**d**) and fMIFL-FPR1-Gi-scFv16 (**e**) indicate local resolution in the range of 2.5-4.3 Å in most map regions.

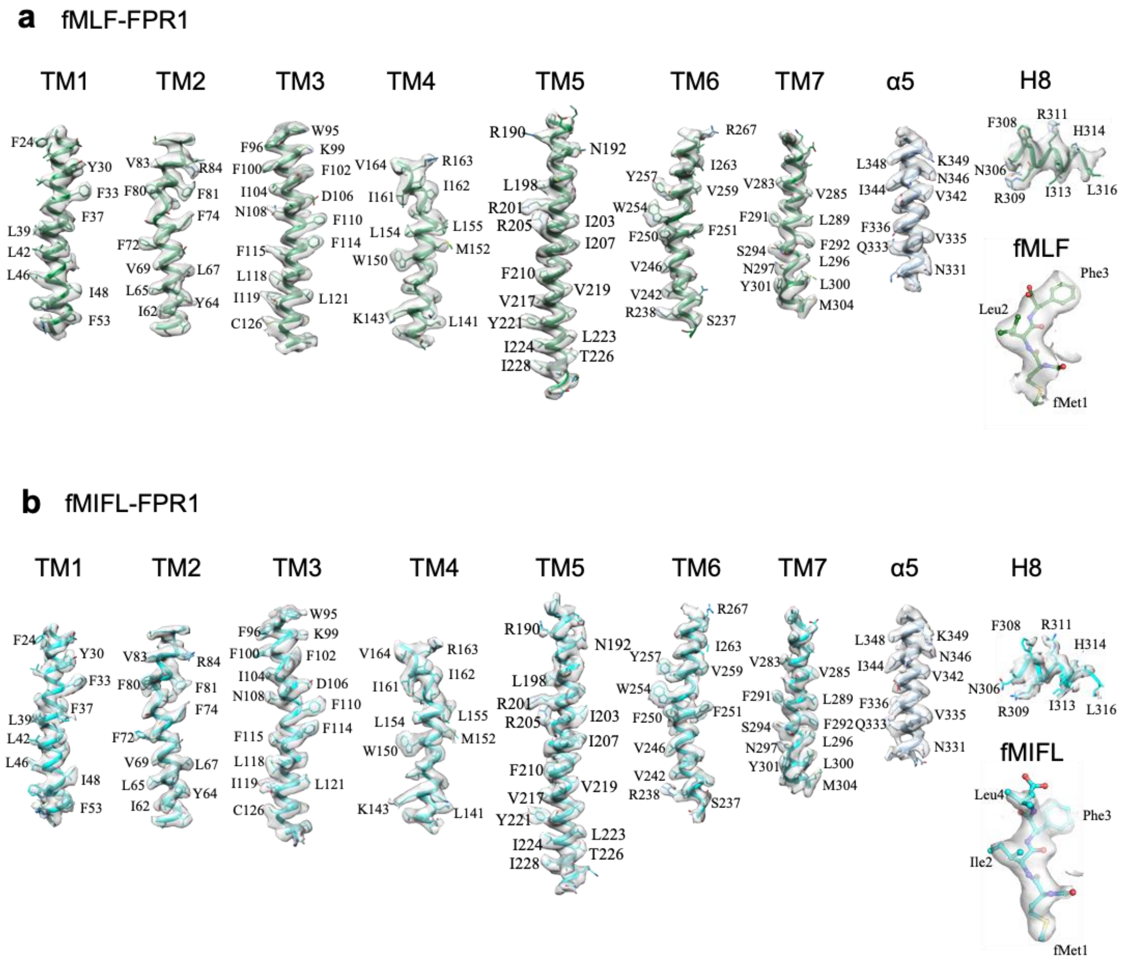

Supplementary Fig. 4. Cryo-EM map and refined structures.

a The Cryo-EM density map and the model of FPR1-fMLF-Gi-scFv16 complex are shown for fMLF, including all transmembrane helices, helix 8, α5 helix of Giα protein. **b** The cryo-EM density map and the model of the FPR1-fMIFL-Gi-scFv16 complex are shown for fMIFL, including all transmembrane helices, helix 8, and α5 helix of Giα protein. The density maps of fMLF and fMIFL are shown at a contour level of 4.0 rmsd.

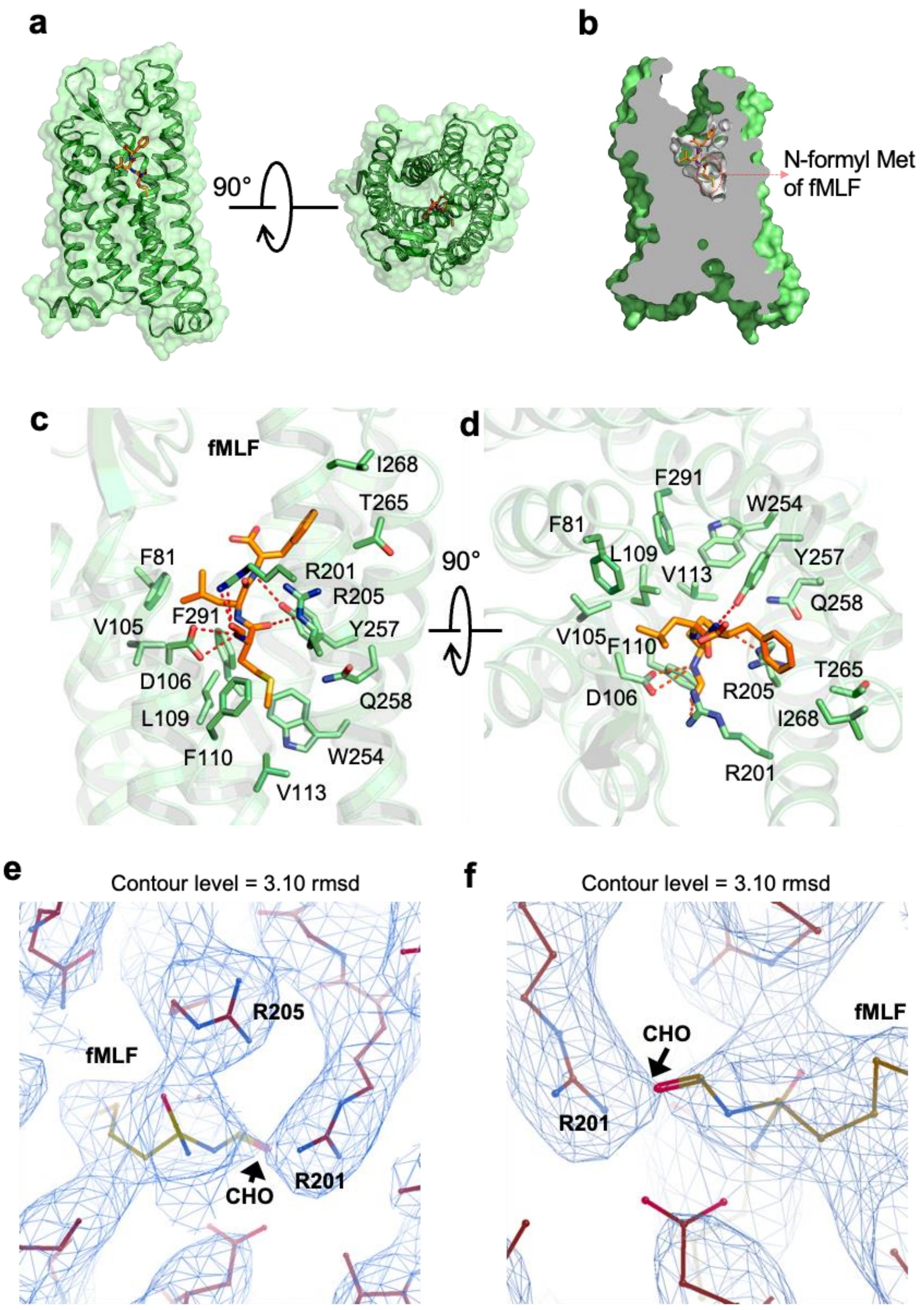

Supplementary Fig. 5. Ligand binding mode of FPR1 to fMLF.

a Side view (left) and extracellular view (right) of the FPR1-fMLF structure. The receptor is shown as surface and cartoon, colored in green. The ligand fMLF is shown as licorice with carbons in orange. **b** Slab view (light grey) of the binding cavity of fMLF in FPR1. fMLF assumes an N-terminus-in pose circled in red dashed line. **c** Side view of the binding pocket of FPR1-fMLF structure. The receptor is shown as cartoon and colored in green. The ligand fMLF is shown as licorice with carbons in orange. The residues of FPR1 within 4.5 Å to the atoms of fMLF are

shown in cyan licorice. **d** Extracellular (top) view of the FPR1-fMLF structure. **e** and **f** Local density map of the ligand fMLF and residues of FPR1 nearby, viewed from two different angles.

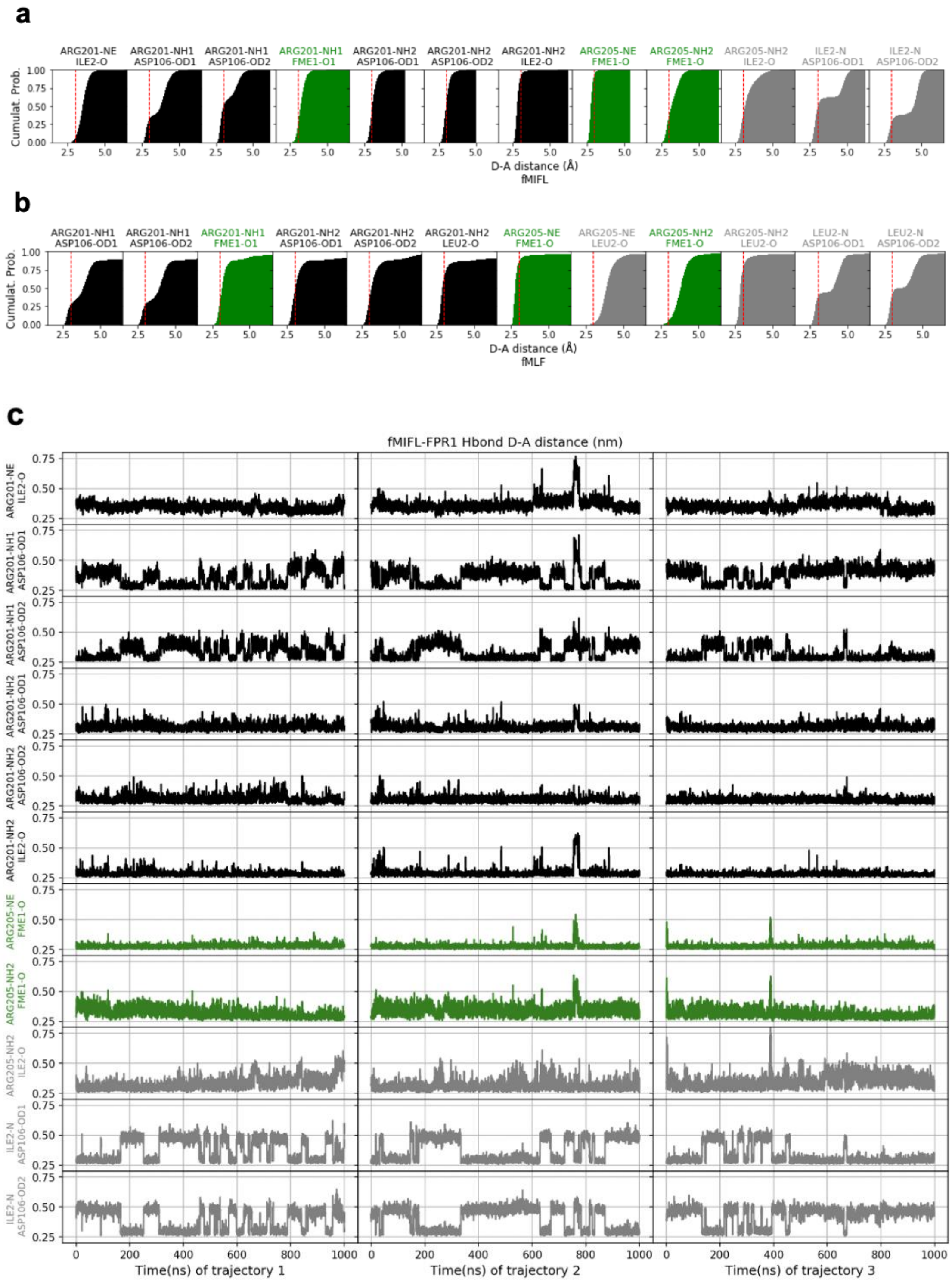

d

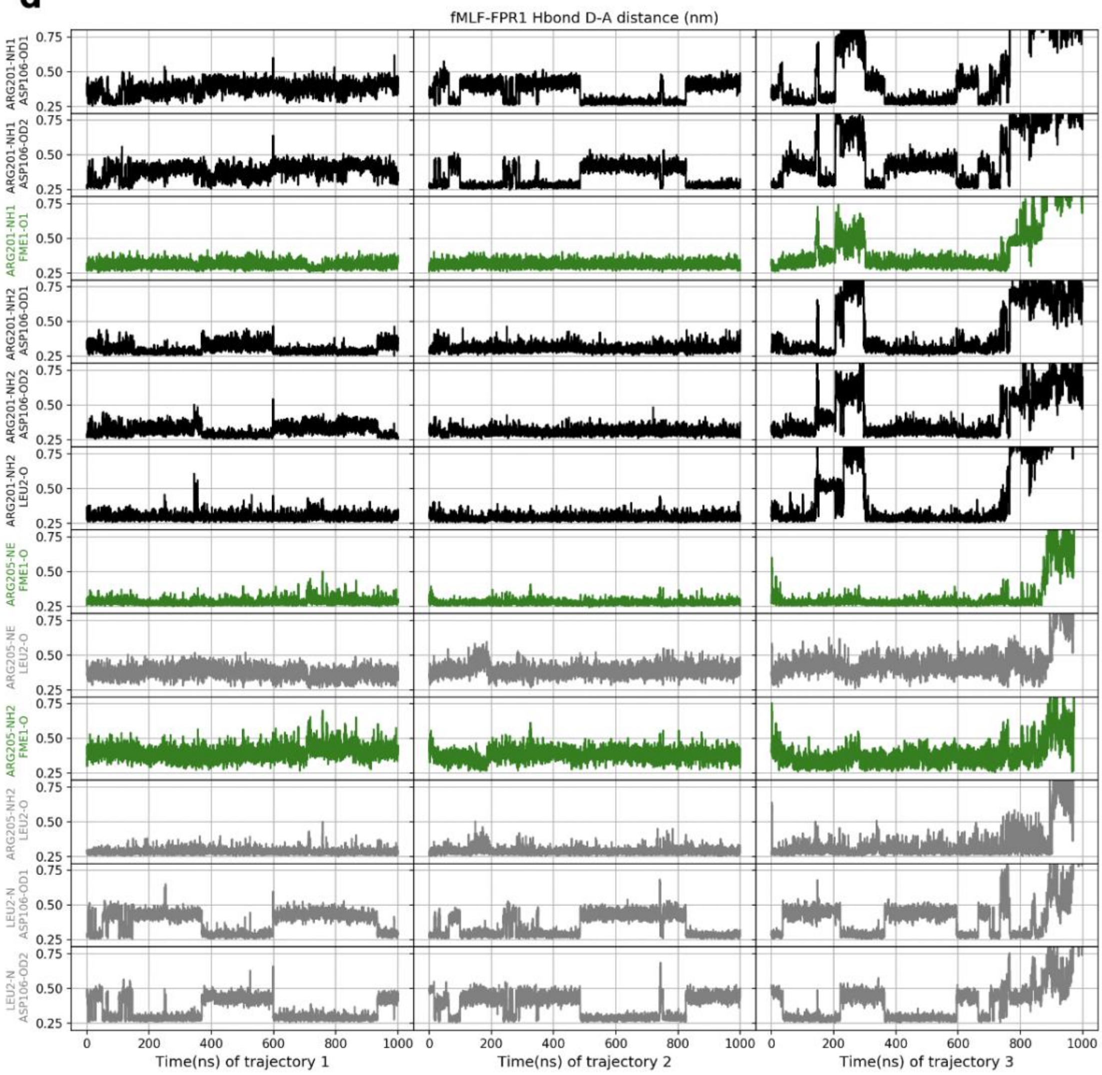

Supplementary Fig. 6. MD simulation of the Cryo-EM models.

Cumulated probability and trajectories of FPR1-Gi-scFv16 bound with fMIFL (**a, c**) and fMLF (**b, d**) from MD simulation (each 1 μs). The distances between fMet and R201$^{5.38}$ and R205$^{5.42}$ are shown in green. The dashed red line indicating the atom distance is 3 Å. R205$^{5.42}$ is found to form hydrogen bond with carbonyl oxygen (FME1-O) of fMet. The formyl oxygen (FME-O1) on fMet1 forms hydrogen bond with R201-NH1. There is also a salt bridge between R201$^{5.38}$-D106$^{3.33}$. Source data are provided as a Source Data file.

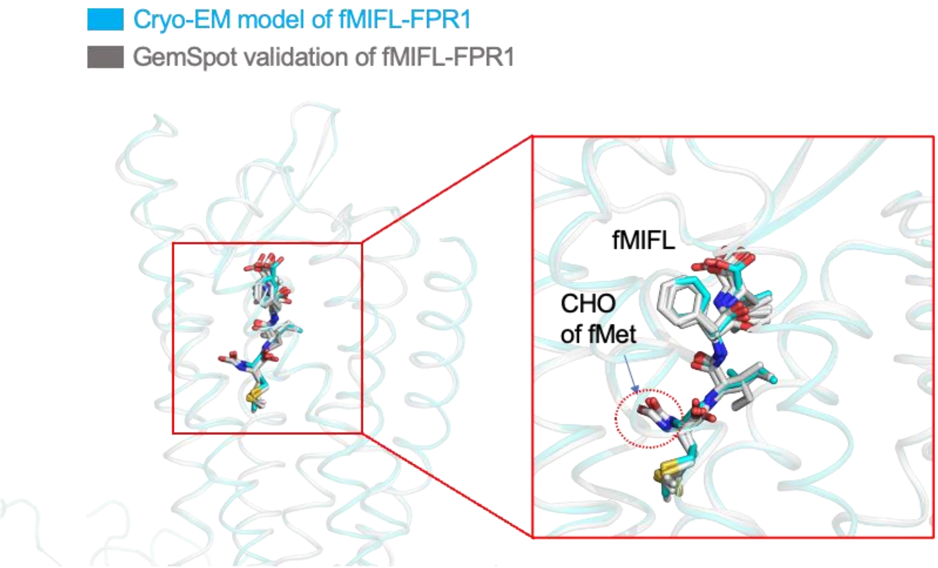

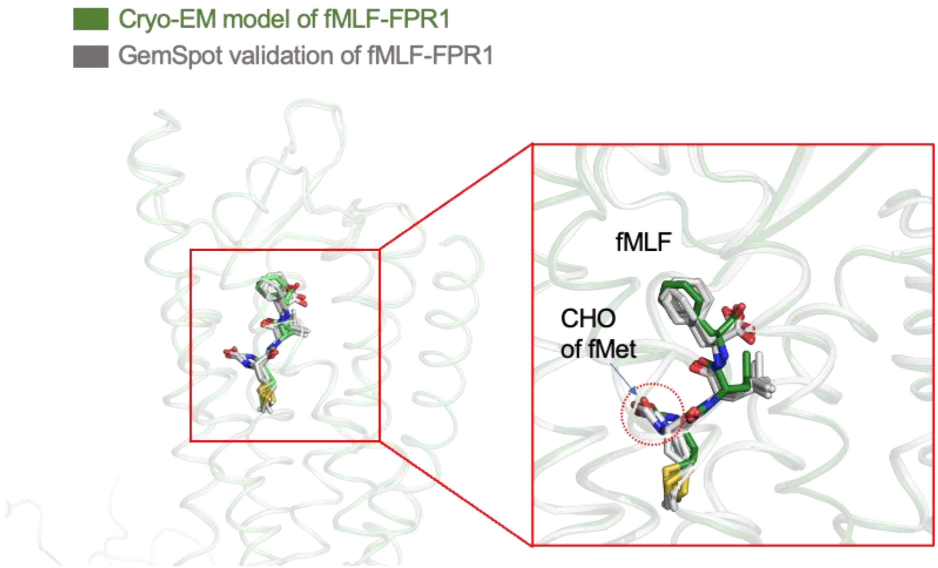

Supplementary Fig. 7. Validation of the model of FPR1-Gi-scFv16 bound with fMIFL (top) and fMLF (bottom) using the GemSpot pipeline. The top-5 high-scored ranked poses (grey) are selected to superimpose with the modeled pose of fMIFL (cyan) and fMLF (green), respectively. The N-formyl group (CHO) is highlighted in red circle. Source data are provided as a Source Data file.

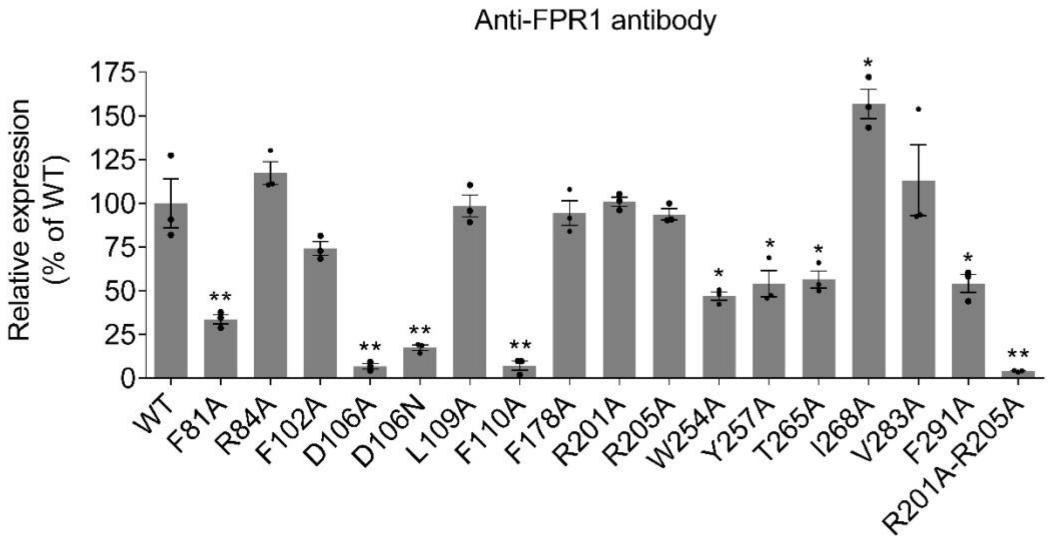

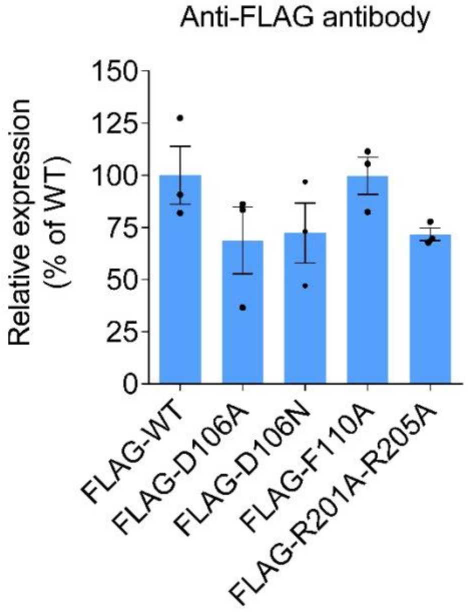

Supplementary Fig. 8. Cell surface expression of FPR1 and its mutants.

Cell surface expression of FPR1 and its mutants were detected using flow cytometry 24 h after transient transfection using an anti-FPR1 mAb labeled with Alexa Fluor® 647 (top panel). Selected receptors were FLAG tagged at the N-termini, expressed and detected with an anti-FLAG antibody (M2, FITC-labeled). Relative expression levels of the mutants are calculated based on WT receptor expression (100%). Data are shown as mean ± SEM of three independent experiments (n = 3). *$P < 0.05$, **$P < 0.01$ (unpaired two-tailed Student's t test, each mutant compared with WT). The exact P values are shown in the Source Data file.

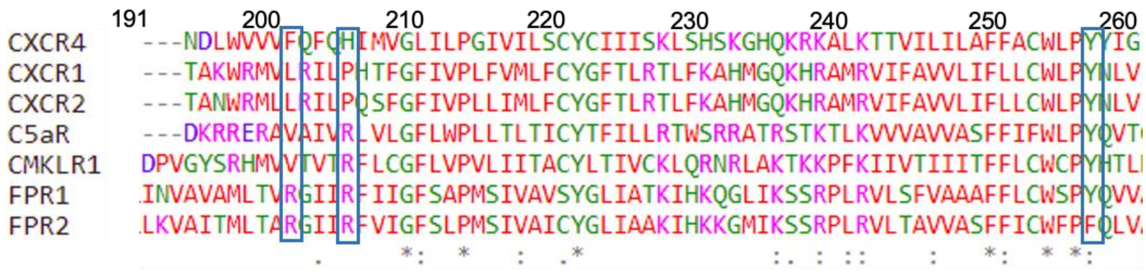

Supplementary Fig. 9. Sequence comparison of the $R^{5.38}XXXR^{5.42}$ motif.

The characterized residues are highlighted in blue square in the multiple sequence alignment. $R201^{5.38}$ is critical in ligand recognition of FPRs, which is not conserved in other GPCRs. $R205^{5.42}$ appears in C5aR and CMKLR1, and these two receptors are phylogenetically closer to FPRs than others. $W257^{6.51}$ is conserved in FPR1 and other class A GPCR receptors, but it is replaced by a phenylalanine (F) in FPR2. Source data are provided as a Source Data file.

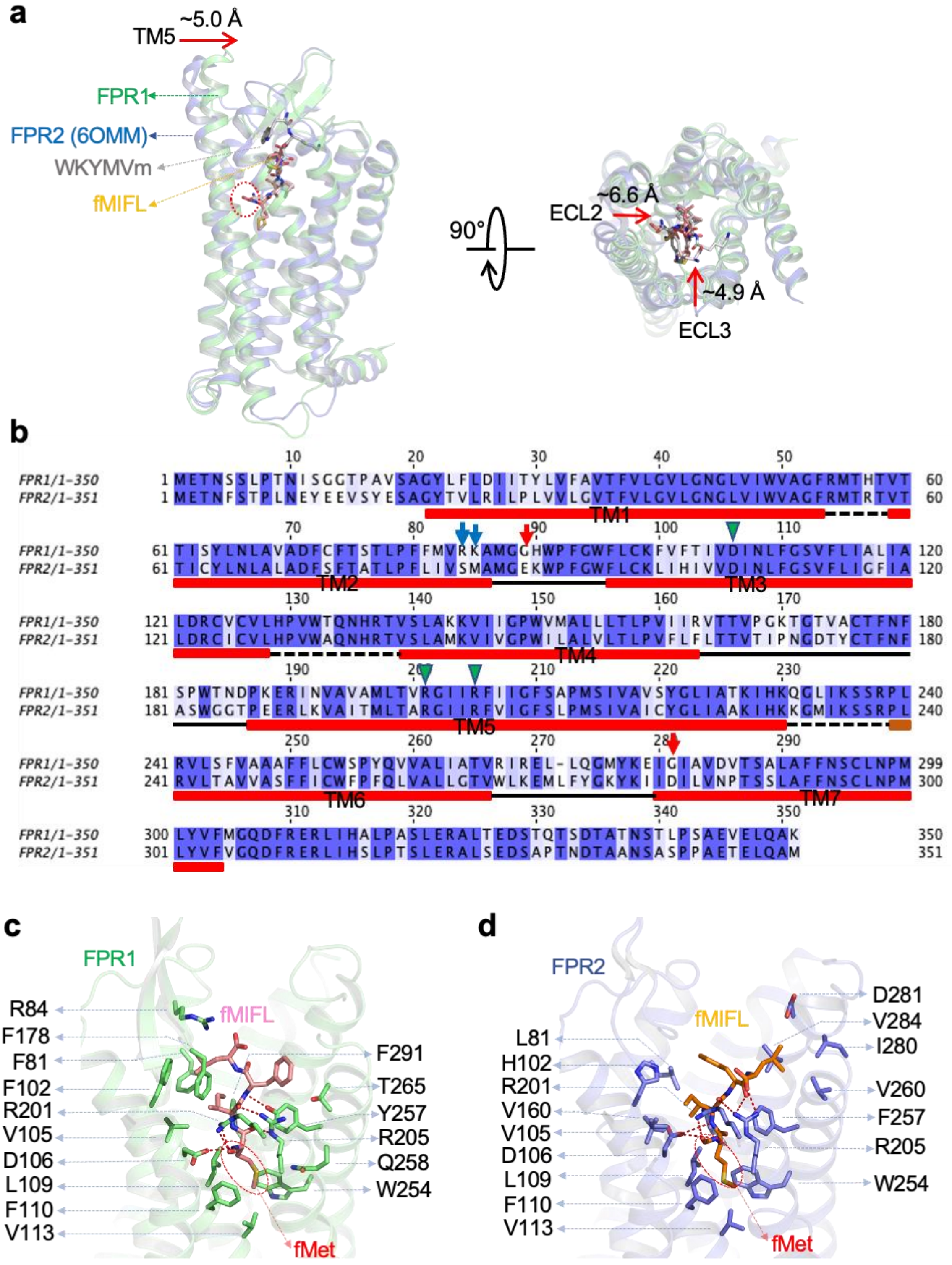

Supplementary Fig. 10. Comparison between FPR1 and FPR2.

(see next page)

Supplementary Fig 10. Comparison between FPR1 and FPR2.

a Side view (left) and top view (right) of the superimposed structures of FPR1 (green) and FPR2 (marine blue, 6OMM), showing an inward shift (red arrows) of the TM5 that reduces the size of the ligand binding pocket of fMIFL (orange) and WKYMVm (grey) in FPR1 (green) compared to FPR2 (marine blue). And the inward movement of both ECL2 and ECL3 in FPR1 makes the open mouth is not as large as FPR2. **b** Sequence alignment of FPR1 and FPR2. The red box, dashed line, and solid line indicates TM, ICL, and ECL domains, respectively. Conserved residues are colored in blue. Basic residues R84, K85 in FPR1 but not conserved in FPR2 are indicated by blue arrows. Acidic residues E89 and D281 in FPR2 but not conserved in FPR1 are indicated by red arrows. D106, R201, and R205 that are critical in ligand recognition are indicated by green triangles. Comparison of FPR1 with FPR2 is shown in **c** and **d**. **c** Side view of the FPR1-fMIFL cryo-EM structure. The receptor is shown as cartoon and colored in grass green. The ligand fMIFL is shown as licorice with carbons in pink. **d** Side view of the represented docking pose of FPR2-fMIFL. FPR2 and fMIFL are show in marine blue cartoon and pink licorice, respectively. The residues of receptor within 4.5 Å to the atoms of the ligand are highlighted in licorice in the binding cavities of FPR1 and FPR2. N-formyl Met of fMIFL is circled in red. The docking result suggests polar interactions between fMIFL and FPR2 involving D106, R201, and R205. Source data are provided as a Source Data file.

Supplementary Table 1. Cryo-EM Data Collection, Model Refinement and Validation Statistics

	FPR1-fMLF	FPR1-fMIFL
**Data collection and processing**		
Magnification	105,000	105,000
Voltage (kV)	300	300
Electron exposure (e–/Å2)	55	49.5
Defocus range (μm)	−1.2 ~−2.0	−1.0 ~−1.8
Pixel size (Å)	0.85	0.85
Symmetry imposed	C1	C1
Initial particle images (no.)	4,310,681	1,690,578
Final particle images (no.)	392,103	230,890
Map resolution (Å)	2.9 Å	2.8 Å
FSC threshold	(0.143)	(0.143)
Map resolution range (Å)	2.5-4.3	2.5-4.3
**Refinement**		
Initial model used (PDB code)	6DDE	6DDE
Model resolution (Å)	3.5	3.5
Model resolution range (Å)	2.5-10	2.4-10
Map sharpening B factor (Å2)	-106.9	-70.5
Model composition		
Non-hydrogen atoms	8951	9005
Protein residues	1138 residues	1139 residues
Ligands	3	4
B factors (Å^2)		
Protein	117.33	63.42
Ligand	126.11	26.33
R.m.s. deviations		
Bond lengths (Å)	0.004	0.003
Bond angles (°)	0.595	0.638
Validation		
MolProbity score	1.66	1.45
Clashscore	6.54	5.04
Rotamer outliers (%)	0	0
Ramachandran plot		
Favored (%)	95.71	96.88
Allowed (%)	4.29	3.12

Supplementary Table 2. Primers used in this study

Primer Name	Primer Sequence (5' to 3')
FPR1as-F	CTGGCTAGCGTTTAAACTTAAGCTTGCACCATGGAG ACAAATTCCTCTCTCCCC
FPR1as-R	TGTGCTGGATATCTGCAGAATTCTCACTTTGCCTGT AACTCCACCTCT
FLAG-FPR1-F1	GCACCATGGACTACAAAGACGATGACGACAAGGAG ACAAATTCCTCTCTCCCCACG
FLAG-FPR1-F2	CTGGCTAGCGTTTAAACTTAAGCTTGCACCATGGAC TACAAAGACGATGAC
F81A-F	CACCTCCACTTTGCCATTCGCAATGGTCAGGAAGG
F81A-R	AATGGCAAAGTGGAGGTGAAACAGAA
R84A-F	GCAAAGGCCATGGGAGGACATTGG
R84A-R	TCCTCCCATGGCCTTTGCGACCATGAAGAATGGCA AAGT
F102A-F	GTTCCTGTGCAAATTCGTCGCAACCATAGTGGACAT CAACTTG
F102A-R	ACGAATTTGCACAGGAACCAGC
D106A-F	GCAATCAACTTGTTCGGAAGTGTCTTCC
D106A-R	CTTCCGAACAAGTTGATTGCCACTATGGTAAAGACG AATTTGCA
D106N-F	AACATCAACTTGTTCGGAAGTGTCTTCC
D106N-R	CTTCCGAACAAGTTGATGTTCACTATGGTAAAGACG
L109/110A-R	TGATGTCCACTATGGTAAAGACGAATTTGC
L109A-F	CTTTACCATAGTGGACATCAACGCATTCGGAAGTGT CTTCC
F110A-F	CTTTACCATAGTGGACATCAACTTGGCAGGAAGTGT CTTCC
F178A-F	AACGGGGACAGTAGCCTGCACTGCCAACTTTTCGC
F178A-R	GGCTACTGTCCCCGTTTTACCAG
R201A-F	GCAGGCATCATCCGGTTCATCATTG
R201A-R	GAACCGGATGATGCCTGCCACCGTCAACATGGCAA
R205A-F	GCATTCATCATTGGCTTCAGCGCAC
R205A-R	GAAGCCAATGATGAATGCGATGATGCCTCTCACCG TCAACA
R201A-R205-F	GCATTCATCATTGGCTTCAGCGCAC
R201A-R205-R	GAAGCCAATGATGAATGCGATGATGCCTGCCACCG
Y257A-F	TTTCTCTGCTGGTCCCCAGCCCAGGTGGTGGC
Y257F-F	TTTCTCTGCTGGTCCCCATTCCAGGTGGTGGC
Y257A/F-R	GGGGACCAGCAGAGAAAAAAGG
T265A-F	GGCCCTTATAGCCGCAGTCAGAATCC
T265A-R	ACTGCGGCTATAAGGGCCACCACC
I268A-F	CCTTATAGCCACAGTCAGAGCCCGTGAGTTATTGCA AG
I268A-R	TCTGACTGTGGCTATAAGGGCCA

V283A-F	CAAAGAAATTGGTATTGCAGCAGATGTGACAAGTGCCCTG
V283A-R	CTGCAATACCAATTTCTTTGTACATGCC
F291A-F	GTGACAAGTGCCCTGGCCGCATTCAACAGCTG
F291A-R	CCAGGGCACTTGTCACATCCA
