# Supplementary material for: Structural basis for recognition of N-formyl peptides as pathogen-associated molecular patterns
Source: Nat Commun. 2022 Sep 5;13:5232. doi: 10.1038/s41467-022-32822-y (PMC9445081; doi:10.1038/s41467-022-32822-y)
Supplement: Supplementary file 2 — Description of Additional Supplementary Files [file 41467_2022_32822_MOESM2_ESM.pdf]

Description of Additional Supplementary Files

File name: Supplementary Data 1

Description: The PDB file of docking complex of MLF-FPR1

File name: Supplementary Data 2

Description: The PDB file of docking complex of tBOC-MLF-FPR1

File name: Supplementary Data 3

Description: The PDB file of docking complex of WKYMVm-FPR1

File name: Supplementary Data 4

Description: The PDB file of docking complex of AG14-FPR1

File name: Supplementary Data 5

Description: The PDB file of docking complex of compound17b-FPR1
