# Supplementary material for: Structural basis for recognition of N-formyl peptides as pathogen-associated molecular patterns
Source: Nat Commun. 2022 Sep 5;13:5232. doi: 10.1038/s41467-022-32822-y (PMC9445081; doi:10.1038/s41467-022-32822-y)
Supplement: Supplementary file 8 — Reporting Summary [file 41467_2022_32822_MOESM8_ESM.pdf]

nature portfolio

Corresponding author(s): Richard D. Ye

Last updated by author(s): Aug 5, 2022

Reporting Summary

Nature Portfolio wishes to improve the reproducibility of the work that we publish. This form provides structure for consistency and transparency in reporting. For further information on Nature Portfolio policies, see our Editorial Policies and the Editorial Policy Checklist.

Statistics

For all statistical analyses, confirm that the following items are present in the figure legend, table legend, main text, or Methods section.

n/a	Confirmed	
☐	☒	The exact sample size (*n*) for each experimental group/condition, given as a discrete number and unit of measurement
☐	☒	A statement on whether measurements were taken from distinct samples or whether the same sample was measured repeatedly
☐	☒	The statistical test(s) used AND whether they are one- or two-sided<br>*Only common tests should be described solely by name; describe more complex techniques in the Methods section.*
☒	☐	A description of all covariates tested
☒	☐	A description of any assumptions or corrections, such as tests of normality and adjustment for multiple comparisons
☐	☒	A full description of the statistical parameters including central tendency (e.g. means) or other basic estimates (e.g. regression coefficient) AND variation (e.g. standard deviation) or associated estimates of uncertainty (e.g. confidence intervals)
☐	☒	For null hypothesis testing, the test statistic (e.g. *F*, *t*, *r*) with confidence intervals, effect sizes, degrees of freedom and *P* value noted<br>*Give P values as exact values whenever suitable.*
☒	☐	For Bayesian analysis, information on the choice of priors and Markov chain Monte Carlo settings
☒	☐	For hierarchical and complex designs, identification of the appropriate level for tests and full reporting of outcomes
☒	☐	Estimates of effect sizes (e.g. Cohen's *d*, Pearson's *r*), indicating how they were calculated

Our web collection on statistics for biologists contains articles on many of the points above.

Software and code

Policy information about availability of computer code

Data collection	The movie stacks with 50 frames were automatically collected using the SerialEM 3.8 software.
Data analysis	Movie stack alignment was done using MotionCor2_1.3.0-Cuda101 (Ref.42);<br>CTF estimate was done using Kai Zhang's Gctf program (Ref. 43), the Bayesian polishing program was used to estimate trajectories of particle motion and the amount of cumulative beam damage;<br>Local resolution was estimated using blocres implemented in cryoSPARC 3.3.1;<br>Particles were auto-picked using Laplacian-of-Gaussian filter in RELION 3.1 (Ref.44);<br>Phenix 1.18.2 was used for dockin all the models into the electron density map, generation of the coordinates and geometry restrains of fMLF, fMIFL and cholestrol, as well as model refinement;<br>Ligand fitting into the electron density map and real space refinement were executed in Coot 0.9.7;<br>Surface coloring of the density map was performed using UCSF Chimera 1.16;<br>MD simulation was performed using GROMACS (version 2020.2);<br>Figures were prepared with PyMOL 2.4.0;<br>Results of cellular assays were analyzed with Prism 6.0.

For manuscripts utilizing custom algorithms or software that are central to the research but not yet described in published literature, software must be made available to editors and reviewers. We strongly encourage code deposition in a community repository (e.g. GitHub). See the Nature Portfolio guidelines for submitting code & software for further information.

Data

Policy information about availability of data

All manuscripts must include a data availability statement. This statement should provide the following information, where applicable:

- Accession codes, unique identifiers, or web links for publicly available datasets
- A description of any restrictions on data availability
- For clinical datasets or third party data, please ensure that the statement adheres to our policy

The datasets generated during the current study are available in the Protein Data Bank (PDB) repository (PDB ID:7EUO, PDB ID:7VFX) and EM Data Bank with EMDB-31323, EMDB-31962. We also used the coordinates of FPR2 cryo-EM structures (PDB ID: 6LW5, PDB ID: 6OMM) for docking analysis.

Field-specific reporting

Please select the one below that is the best fit for your research. If you are not sure, read the appropriate sections before making your selection.

☒ Life sciences ☐ Behavioural & social sciences ☐ Ecological, evolutionary & environmental sciences

For a reference copy of the document with all sections, see nature.com/documents/nr-reporting-summary-flat.pdf

Life sciences study design

All studies must disclose on these points even when the disclosure is negative.

Sample size	Sample size of 3 or more (Fig. 5 and Supplementary Fig. 8) was chosen in functional assays. Based on previous experience and the guidelines for statistics in Prism [https://www.graphpad.com/guides/prism/latest/statistics/stat_sample_size_for_which_values_o.htm], 3 independent experiments are sufficient to overcome sample variations in the highly reproducible cAMP assay with the commercially available kit.
Data exclusions	No data exclusion was applied to this study.
Replication	Where applicable (Fig. 5 and Supplementary Fig. 8), data are shown as mean ± SEM of at least three independent experiments, each with duplicates. The duplicate measurement is in order to eliminate the systemic error because of the high reproducibility of commercial kits.
Randomization	In Cryo-EM data collection and data processing, the particles are randomly picked. In addition, we have described the Gene ID of FPR1 (which has several identified SNPs in human) in the manuscript (Gene ID: 2357).
Blinding	As this is not a population based study, blinding is not applicable for this study.

Reporting for specific materials, systems and methods

We require information from authors about some types of materials, experimental systems and methods used in many studies. Here, indicate whether each material, system or method listed is relevant to your study. If you are not sure if a list item applies to your research, read the appropriate section before selecting a response.

Materials & experimental systems

n/a	Involved in the study	
☐	☒	Antibodies
☐	☒	Eukaryotic cell lines
☒	☐	Palaeontology and archaeology
☒	☐	Animals and other organisms
☒	☐	Human research participants
☒	☐	Clinical data
☒	☐	Dual use research of concern

Methods

n/a	Involved in the study	
☒	☐	ChIP-seq
☐	☒	Flow cytometry
☒	☐	MRI-based neuroimaging

Antibodies

Antibodies used	Alexa Fluor® 647-labeled anti-FPR1 antibody (5F1; Becton Dickinson, Cat #565623; 1:50 diluted by HBSS buffer);<br>FITC-labeled anti-FLAG antibody (M2; Sigma, Cat #F4049; 1:50 diluted by HBSS buffer).
Validation	All antibodies were commercially purchased and validation reports are available on the supplier websites: Validation for Alexa Fluor® 647-labeled anti-FPR1 antibody: https://www.bdbiosciences.com/content/dam/bdb/products/global/reagents/flow-cytometry-reagents/research-reagents/single-color-antibodies-ruo/565623_base/pdf/565623.pdf; Validation for FITC-labeled anti-FLAG antibody: https://www.sigmaaldrich.com/specification-sheets/128/312/F4049-BULK_________SIGMA_____.pdf

Eukaryotic cell lines

Policy information about cell lines

Cell line source(s)	Spodoptera frugiperda (Sf9) insect cells: Invitrogen; Trichoplusia ni Hi5 insect cells (Invitrogen); HeLa cells (ATCC).
Authentication	None of the cell lines have been authenticated.
Mycoplasma contamination	The cell lines were negative for mycoplasma contamination.
Commonly misidentified lines (See ICLAC register)	No commonly misidentified cell lines were used.

Flow Cytometry

Plots

Confirm that:

- [ ] The axis labels state the marker and fluorochrome used (e.g. CD4-FITC).
- [ ] The axis scales are clearly visible. Include numbers along axes only for bottom left plot of group (a 'group' is an analysis of identical markers).
- [ ] All plots are contour plots with outliers or pseudocolor plots.
- [ ] A numerical value for number of cells or percentage (with statistics) is provided.

Methodology

Sample preparation	FPR1 and its mutants were transiently expressed in HeLa cells for 24 h. The cells were incubated on ice for 1 h with Alexa Fluor® 647-labeled anti-FPR1 antibodies (Becton Dickinson, Cat #565623; 1:50 diluted by HBSS buffer). The N-terminal FLAG-tagged WT and mutant receptors were detected with a FITC-labeled anti-FLAG antibody (M2; Sigma, Cat #F4049; 1:50 diluted by HBSS buffer). After washing, cell fluorescence was detected by the Accuri C6 Plus flow cytometer (Becton Dickinson, Franklin Lakes, NJ, USA).
Instrument	Accuri C6 Plus flow cytometer (Becton Dickinson, Franklin Lakes, NJ, USA)
Software	Data were analyzed with Prism 6.0.
Cell population abundance	For each measurement, 10,000 cell events were collected within the gated population.
Gating strategy	Gating was determined by fluorescence intensities under different spectra (Alexa Fluor® 647 and FITC, respectively).

- [ ] Tick this box to confirm that a figure exemplifying the gating strategy is provided in the Supplementary Information.
